# Supplementary material for: Cross-sectional experimental assessment of pain modulation as part of multidimensional profiling of people with cervicogenic headache: protocol for a feasibility study
Source: BMJ Open. 2024 Jun 18;14(6):e074743. doi: 10.1136/bmjopen-2023-074743 (PMC11191774; doi:10.1136/bmjopen-2023-074743)
Supplement: Supplementary data [file bmjopen-2023-074743supp001.pdf]

SUPPLEMENTARY FILE

Supplement 1 – Checklist participant recruitment

The criteria that will be used to in- and exclude participants are summarized in table S1.

Table S1. Summary of inclusion- and exclusion criteria for participants with CeH.

**Inclusion**	Caucasian males, females between 18-55 years
	Dutch-speaking
	Fulfilment of the diagnostic criteria for secondary CeH conform the ICHD-3 or CHISG (1,2)
	Diagnostic conformation by a neurologist
	Normal cognitive capacity (Mini Mental State Examination test score of 30).
	Willing to participate
**Exclusion**	Any other headache type or headache-related disorder
	Participation in another study related to headache
	Pregnancy
	Smoking
	First headache onset > 50 years
	Spinal surgery, head/neck trauma, headache-related physiotherapy ≤ 4 weeks prior the study
	Post-dural punction 2 weeks prior to the study
	Cognitive limitations (Mini Mental State Examination test score < 30)
	Confounding pathologies
	- Musculoskeletal: hernia, disk prolapse, congenital spinal deviations, TMD, hypermobility (Beighton ≥ 4)
	- Neurological: MS, Parkinson, epilepsy, CVA, myelopathy, myopathy, Benign Paroxysmal Position Vertigo, neurodegeneration, meningitis, encephalitis, dystonia, visual disorder
	- Endocrine - System: FM, CFS, rheumatoid arthritis, infectious/inflammatory diseases, sinusitis
	- Vascular: dysregulation blood pressure, postural hypotension, vertebra-basilar symptoms
	- Psychiatric
	- Medication-overuse: ergotamine, NSAID's, opioids, acetylsalicylic acid, triptans, simple analgesics (> 10 days/month for > 3 months)
	- Withdrawal of alcohol, caffeine or medication
	- Other: orthodontics, sleep apnoea

ICHD = International Classification Headache Disorders; CHISG = Cervicogenic Headache International Study Group Diagnostic; NSAID = Non Steroid Anti Inflammatory; MS = Multiple Sclerosis; CVA = Cerebro-Vascular Accident; FM = Fibromyalgia; CFS = Chronic Fatigue Syndrome; TMD = Temporo-mandibular disorder.

Supplement 2 – Time schedule

The time schedule for the feasibility study is visualized via figure S1.

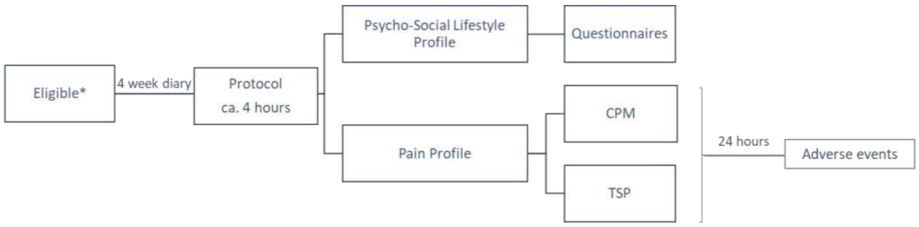

Figure S1. Visualisation of the time schedule for the feasibility study (* = estimated time to recruit at least 12 people with CeH = 3 months based on previous experience; CPM = Conditioned Pain Modulation; TSP = Temporal Summation of Pain).

Supplement 3 – Conditioned pain modulation – widespread hyperalgesia

Figure S2 provides a summary of the second CPM protocol.

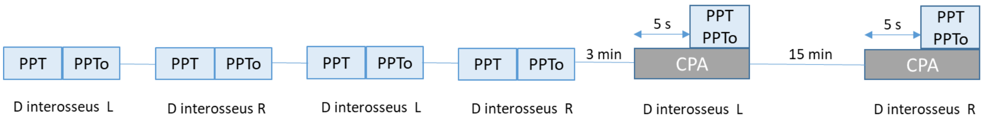

Figure S2. Visualisation of the experimental protocol regarding CPM (PPT = Pressure Pain Threshold (kPa/cm²); PPTo = Pressure Pain Tolerance (kPa/cm²); D = Dorsal; L = Left; R = Right; CPA = Cuff Pressure Algometry non-dominant calf muscle (kPa); s = seconds; min = minutes).

Test stimulus: Mechanical pressure pain thresholds and tolerance

Measurements will be performed 15 minutes after the CPM with the testing stimulus applied at the suboccipital muscles. Pressure pain threshold (PPT) and pressure pain tolerance (PPTo) (kPa/cm²) will be measured at the left and right muscle belly of the first dorsal interosseous muscle using an electronic pressure algometer (Somedic AB, Stockholm, Sweden) (3,4). Pressure will be perpendicularly applied directly on the midpoint of the muscle belly, starting at 0 to maximal 1000 kPa, using a 1 cm² probe with a slope of 30 kPa/s. (5,6). Participants will be instructed to push the stop-button when the sensation of pressure first elicits pain (PPT), and when the maximum amount of pressure that could be tolerated is reached (PPTo).Intrarater reliability of the first dorsal interosseous muscle is excellent in healthy participants (ICC 0.91) (3).

Two repeated measurements at baseline, and two measurements parallel with the conditioning stimulus will be performed (figure S2). Averages of the extracted parameters will be used for further analysis (7).

Conditioning stimulus: Cuff pressure algometry

Cuff pressure tolerance (kPa) will be measured at the non-dominant lower leg. A cuff (Nocitech, Aalborg University, Denmark) will be placed around the bare non-dominant calf muscle, mounted with 8-cm distance between its upper border and the tibial tuberosity (8,9). Upper and lower borders of the cuff will be marked on the skin to ensure the cuff does not move between stimulations. Pressure tolerance will be determined through the maximal amount of pressure that can be tolerated. Intensity of the cuff pressure for the conditioning stimulus will be predefined as 70% of the pressure tolerance on the non-dominant leg (9,10). Computer-controlled cuff algometry (LabBench® CPAR+ instrument) shows an excellent intra-rater reliability (ICC 0.89) in healthy subjects (11,12). Test-retest reliability is good (ICCs 0.74 – 0.87) in healthy subjects (8).

Conditioned pain modulation (CPM)

CPM will be assessed 3 minutes after determining cuff pressure tolerance. The pressure (conditioning stimulus) will be kept constant throughout the CPM protocol (9,10). Five seconds after inflation of the cuff, PPT and PPTo will be reassessed as described above. Participants will be informed that the conditioning stimulus will be moderately painful, but that they should focus their attention on the test stimulus. The procedure described above will be sequentially and twice executed, i.e. once with the PPT-measurement at the left first dorsal interosseous muscle, and once with the PPT-measurement at the right first dorsal interosseous muscle as test stimulus. A 15-minute-interval will be provided between the CPM-measurements since CPM has a short lasting (< 15 minutes) hypoalgesic effect (figure S2) (14,15).

At the moment, there is no consensus on a normal CPM effect. We propose a method described by Vaegter et al. (2016) to categorize the CPM response as normal or impaired based on the within-subject coefficient of variation (= within-subject SD/within-subject mean) in PPT (7). Participants will be profiled as having an impaired CPM if the CPM response is less than or equal to the normal within-subject coefficient of variation in PPT between 2 repeated assessments, and as having normal CPM if the CPM response is greater than the normal variation plus the upper limit of the 95% confidence interval (7).

Supplement 4 – Summary of the primary outcomes

Table S2 provides a summary of the primary outcomes to estimate feasibility.

Table S2. Summary of the adapted feasibility metrics and objectives (15,16).

Metric	Objectives	Outcome
**Process**	Determine recruitment and/or refusal rates per month	Percentage
Assess feasibility of processes key for future studies	Determine premature session endings and reasons(s) (e.g. pain, discomfort)	Percentage
	Operational feasibility (User Experience Questionnaire on usefulness, satisfaction, ease of learning, ease of use, and user experience after testing) (17)	Scores on a 7-point Likert scale converted to values ranging between < -3 (bad evaluation) and > 3 (extremely good evaluation)
**Resource**	Demonstrate appropriate eligibility criteria	Percentage and Qualitative
Assess time and resource problems that might occur in future studies	Estimate barriers/refusals to participation	Percentage and Qualitative
	Estimate access to/cost of equipment, space, time	Estimate cost/participant (i.e. staff, equipment, facility overheads)
	Clinician training needs and competence	Staff training requirements (time, frequency)
**Management**	Estimate equipment usage (e.g. availability)	Qualitative
Assess potential human and data management problems	Determine processing time for data collection	Time to mail data collection, time to complete outcome assessment
	Data collection and analysis – software appropriateness	Qualitative (identify requirements)
**Scientific**	Estimate challenges perceived/experienced by researcher	Qualitative
Assess safety, burden, data collection and response	Determine adverse events and safety procedure (e.g. local pain)	Percentage and Qualitative
	Burden of research	Percentage and Qualitative (feedback from participants before/during/after measurements)
	Determine appropriateness of target group	Qualitative (feedback from participants)
	Determine acceptability to participants	Qualitative (feedback from participants)
	Estimate outcome measures SD to determine future sample size	Statistical analysis of CPM (Pain Profile) to estimate future sample size

CPM = Conditioned Pain Modulation; SD = Standard Deviation.

References

1. Headache Classification Committee of the International Headache Society (IHS) The International Classification of Headache Disorders, 3rd edition. Cephalalgia 2018;38(1):1-211.
2. Fredriksen TA, Antonaci F, Sjaastad O. Cervicogenic headache: too important to be left un-diagnosed. J Headache Pain 2015;16:6.
3. Chesterton LS, Sim J, Wright CC, et al. Interrater reliability of algometry in measuring pressure pain thresholds in healthy humans, using multiple raters. Clin J Pain 2007;23(9):760-6.
4. Bogduk N. The neck and headaches. Neurol Clin 2014;32(2):471-87.
5. Alburquerque-Sendín F, Madeleine P, Fernández-de-Las-Peñas C, et al. Spotlight on topographical pressure pain sensitivity maps: a review. J Pain Res 2018;11:215-25.
6. Mingels S, Dankaerts W, van Etten L, et al. Exploring multidimensional characteristics in cervicogenic headache: Relations between pain processing, lifestyle, and psychosocial factors. Brain Behav 2021;11(10):e2339.
7. Vaegter HB, Graven-Nielsen T. Pain modulatory phenotypes differentiate subgroups with different clinical and experimental pain sensitivity. Pain 2016;157(7):1480-8.
8. Graven-Nielsen T, Vaegter HB, Finocchietti S, et al. Assessment of musculoskeletal pain sensitivity and temporal summation by cuff pressure algometry: a reliability study. Pain 2015;156(11):2193-202.
9. Cummins TM, Kucharczyk MM, Graven-Nielsen T, et al. Activation of the descending pain modulatory system using cuff pressure algometry: Back translation from man to rat. Eur J Pain 2020;24(7):1330-8.
10. Hoegh M, Petersen KK, Graven-Nielsen T. Effects of repeated conditioning pain modulation in healthy volunteers. Eur J Pain. 2018;22(10):1833-43.
11. Inventors'Way ApS. Cuff Pressure Algometry [online]. 2023. https://nocitech.com (accessed April 15th 2023).
12. Kvistgaard Olsen J, Fener DK, Wæhrens EE, et al. Reliability of Pain Measurements Using Computerized Cuff Algometry: A DoloCuff Reliability and Agreement Study. Pain Pract 2017;17(6):708-17.
13. Vaegter HB, Handberg G, Graven-Nielsen T. Similarities between exercise-induced hypoalgesia and conditioned pain modulation in humans. Pain 2014;155(1):158-67.
14. Vaegter HB, Handberg G, Jørgensen MN, et al. Aerobic exercise and cold pressor test induce hypoalgesia in active and inactive men and women. Pain Med 2015;16(5):923-33.
15. Learmonth YC, Motl RW. Important considerations for feasibility studies in physical activity research involving persons with multiple sclerosis: a scoping systematic review and case study. Pilot Feasibility Stud 2018;4:1.
16. Thorpe N, Harniess P, Main E, et al. Feasibility, safety and acceptability of select outcome measures in a physiotherapy study protocol for boys with haemophilia. Pilot Feasibility Stud 2021;7(1):105.
17. Laugwitz B. Construction and Evaluation of a User Experience Questionnaire. HCI and Usability for Education and Work. Heidelberg: Springer Berlin Heidelberg 2008
