# Supplementary material for: Cross-sectional experimental assessment of pain modulation as part of multidimensional profiling of people with cervicogenic headache: protocol for a feasibility study
Source: BMJ Open. 2024 Jun 18;14(6):e074743. doi: 10.1136/bmjopen-2023-074743 (PMC11191774; doi:10.1136/bmjopen-2023-074743)
Supplement: Author's manuscript [file bmjopen-2023-074743.draft_revisions.pdf]

BMJ Open is committed to open peer review. As part of this commitment we make the peer review history of every article we publish publicly available.

When an article is published we post the peer reviewers' comments and the authors' responses online. We also post the versions of the paper that were used during peer review. These are the versions that the peer review comments apply to.

The versions of the paper that follow are the versions that were submitted during the peer review process. They are not the versions of record or the final published versions. They should not be cited or distributed as the published version of this manuscript.

BMJ Open is an open access journal and the full, final, typeset and author-corrected version of record of the manuscript is available on our site with no access controls, subscription charges or pay-per-view fees (http://bmjopen.bmj.com).

If you have any questions on BMJ Open's open peer review process please email info.bmjopen@bmj.com

BMJ Open

Assessment of Endogenous Pain Modulation as part of Multidimensional Profiling of People with Cervicogenic Headache: A Protocol for a Feasibility Study

Journal:	*BMJ Open*
Manuscript ID	bmjopen-2023-074743
Article Type:	Protocol
Date Submitted by the Author:	17-Apr-2023

Complete List of Authors:	Mingels, Sarah; Katholieke Universiteit Leuven, Department of Rehabilitation Sciences; Hasselt University<br>Granitzer, Marita; Hasselt University<br>Schmid, Annina ; University of Oxford, Nuffield Department of Clinical Neurosciences; The University of Queensland, School of Health and Rehabilitation Sciences<br>Graven-Nielsen, Thomas ; Aalborg University<br>Dankaerts, Wim ; KU Leuven

Keywords:	Feasibility Studies, Chronic Pain, Neurological pain < NEUROLOGY, Risk Factors

SCHOLARONE™
Manuscripts

Assessment of Endogenous Pain Modulation as part of Multidimensional Profiling of People with Cervicogenic Headache: A Protocol for a Feasibility Study

Sarah Mingels[1,2*], Marita Granitzer[2], Annina Schmid[3], Thomas Graven-Nielsen[4], and Wim Dankaerts[1]

[1] Musculoskeletal Research Unit, Department of Rehabilitation Sciences, Faculty of Kinesiology and Rehabilitation Sciences, Leuven University, Leuven, Belgium

[2] REVAL Rehabilitation Research Centre, Biomedical Research Institute, Faculty of Rehabilitation Sciences, Hasselt University, Hasselt, Belgium

[3] Nuffield Department of Clinical Neurosciences, University of Oxford, Oxford, United Kingdom

[4] Center for Neuroplasticity and Pain (CNAP), Department of Health Science and Technology, Aalborg University, Aalborg, Denmark

* corresponding author: sarah.mingels@kuleuven.be

For peer review only

Protocol version: 1

Protocol date: April 15th 2023

ABSTRACT

Background. An endogenous pain modulation profile, reflecting anti-nociceptive and pro-nociceptive mechanisms, may help to direct management by targeting the involved pain-mechanism. For individuals with cervicogenic headache (CeH) the characteristics of such profiles were never investigated. However, the individual nature of experiencing pain demands profiling within a multidimensional framework including psycho-social-lifestyle characteristics. The objective of the current protocol is to assess the pain modulation profile, which includes psycho-social-lifestyle characteristics among people with CeH.

Methods. A protocol is described to map pain modulation profiles in people with CeH. A cross-sectional non-randomized design will be used to assess feasibility of mapping these profiles. The pain modulation profile is composed based on results on the Depression, Anxiety, Stress Scale (DASS-21), Pittsburgh Sleep Quality Index (PSQI), Headache Impact Test (HIT-6), and on responses to temporal summation of pain (pinprick), conditioned pain modulation, and widespread hyperalgesia (mechanical pressure pain threshold and cuff algometry).

Discussion. The objective is a study protocol in which people with CeH can be individually profiled based on their pain modulation profile. Conducting a feasibility study, evaluating this protocol, is warranted before further developing a stepwise pathway of care.

ARTICLE SUMMARY

- An innovative protocol is developed to assess facilitated pain processing in people with cervicogenic headache
- Multiple dimensions (pain processing, psycho-social-lifestyle) of cervicogenic headache will be explored
- No biomarkers exist to determine facilitated pain processing. The pain status can only be estimated through proxy measures such as the conditioned pain modulation and widespread hyperalgesia

For peer review only

1. INTRODUCTION

Cervicogenic headache (CeH) is a secondary headache attributed to dysfunctions of the cervical spine (1). Underdiagnosed and undertreated, CeH might evolve into a chronic state, increasing the odds of absenteeism and disability (2,3). It is generally accepted to adopt a multidimensional approach in the management of pain (4,5) Interestingly though, such an approach is not recommended when managing people with CeH (6). Results from a Delphi-study indicated that lifestyle advice, pain education, and cognitive therapy were considered not to be relevant in the management of CeH (6). Currently, the non-pharmacological management of CeH mainly focusses on targeting the musculoskeletal dysfunctions of the (upper) cervical spine (6-10). However, a meta-analysis with pooled outcome parameters showed inconsistent results of such management on headache intensity, frequency, and related disability (11). Although non-pharmacological interventions may play an important role in managing CeH, there is currently limited scientific evidence to fully support these interventions (11). It has been stated previously that some therapeutic interventions are not appropriate for all people with CeH (12).

When CeH is merely mediated by a peripheral nociceptive source (i.e. musculoskeletal dysfunction of the upper-cervical spine), also known as bottom-up source, this could be managed by addressing the dysfunction through manual therapy and/or specified exercises (13). Such management is likely to be inefficient if signs of facilitated central pain mechanisms are present. In such state, exclusively aiming at the peripheral source might act as a nociceptive stimulus maintaining facilitated central pain processing (14,13) The pathophysiology of CeH can thus generally be explained by (a) CeH caused by an exclusive peripheral input, or (b) CeH caused by peripheral input and maintained by sensitization processes. It has been argued that characterizing the involved dominant central pain mechanism might provide valuable information to increase therapy efficacy (15). Central pain mechanisms such as pro- or antinociceptive mechanisms (16), have however not extensively been examined in people with CeH. Results from a study on pain processing, showing lower extra-cephalic and cephalic pressure pain thresholds in people with CeH compared to healthy controls, might indicate a dysfunctional central pain mechanism (17,18).

Dynamic protocols, to evoke inhibition or facilitation of pain, have been designed to evaluate endogenous pain modulation, and to define a pro- or antinociceptive central pain mechanism. Protocols to assess pain inhibition evaluate the conditioned pain modulation (CPM) paradigm, and pain facilitation assess temporal summation of pain (TSP) (16). The term CPM was created for psychophysical protocols that explore diffuse noxious inhibitory control in humans (19). The latter manifests as the inhibition of wide-dynamic-range neuronal activity by descending adrenergic pathways (19). CPM is measured by comparing pain induced by a test stimulus, with pain induced by the same test stimulus either during (parallel) or after (sequential) a conditioning stimulus. TSP is a

For peer review only - http://bmjopen.bmj.com/site/about/guidelines.xhtml

nociceptive mediated process which is considered to reflect advanced spinal synaptic facilitation in the dorsal horn (i.e. behavioural correlate of wind-up) (20,21). TSP is a measured by comparing pain ratings between a single noxious stimulus and repeated equal-intensity noxious stimuli at a specific frequency. A third proxy to examine facilitated central pain mechanisms is widespread hyperalgesia (22).

Assessing endogenous pain modulation and phenotyping (23-25) in people with CeH within a multidimensional framework is needed in the context of therapy-unresponsiveness. Despite the well-known pathophysiology of CeH, the number of non-responders to non-pharmacological non-invasive therapy amounts to 25%, and self-reported effectiveness of manual therapy is rated as 36% (26,27). Such therapy-unresponsiveness has in other musculoskeletal disorders been related to inadequate health literacy, neural sensitivity, or augmented pain processing in the central nervous system (26, 27). Therefore, a Pain Modulation Profile (PMP) needs to be composed. The PMP includes, besides measurements to analyse central pain mechanisms, also measurements of potential influential factors (e.g. demographic, psycho-social, lifestyle) that can influence such mechanisms. These factors might explain some of the inter-individual variability in pain perception, and therefore, possibly also play a role in CPM (28). Nociceptive inputs activate complex interactions among cortical regions that are also active in cognitive, emotional, and reward functions. These regions influence serotonergic and noradrenergic descending pain modulatory systems bimodally via processes between the periaqueductal grey, rostral ventromedial medulla and pontine noradrenergic nuclei, ultimately facilitating or inhibiting nociceptive input. Descending pain modulatory pathways can therefore be stimulated from the top down, i.e. from the brain to brainstem, by psycho-social interventions (29-31). Further, also lifestyle factors can influence pain processing. Preliminary evidence is provided that an active lifestyle could reduce spinal nociception (i.e. nociceptive flexion reflex) in healthy adults (30). Animal studies indicated that regular physical activity influences central cellular processes (i.e. decrease neuronal excitability, alter neuro-immune signalling, increase release of endogenous opioids and serotonin in the descending pain modulatory pathways) involved in dysregulation of endogenous pain modulatory system, and development of chronic pain (32-34). Additionally, one night of total sleep deprivation impaired descending pain modulatory pathways, facilitated spinal neuronal excitability, and facilitated peripheral pain mechanisms in heathy participants (35).

The objective of the current paper is to describe a study protocol which aims to feasibly assess the PMP among people with CeH. The advantage of determining such PMP is that it offers the possibility to address the involved pain-mechanism(s) within a multidimensional framework.

For peer review only - http://bmjopen.bmj.com/site/about/guidelines.xhtml

2. METHODOLOGY

2.1 Design

A first step toward achieving the objective was to describe a protocol to feasibly assess PMPs among participants with CeH. The next step will be to conduct a feasibility study. The protocol (i.e. eligibility criteria, set-up, analyses of outcomes) will be adapted based on the findings and feedback gathered from the feasibility study. Relevant adaptations will be communicated with the principal investigator (prof. W Dankaerts) and the Ethical Committee Research UZ / KU Leuven.

The measurements and instruments were selected based on a literature review, and after consensus with an expert panel (n = 5) consisting of experts in neurophysiology (MG), neuroscience (AS), biomedical Science, engineering and medical science (TGN), and musculoskeletal rehabilitation (WD, SM) (October 2022 – February 2023).

A description of the measurements, outcomes, instruments, and procedure is outlined below (Table 1 and paragraph 2.5). Enrolled participants will complete the protocol, and will be profiled based on their PMP. This profile comprises a Pain Profile and Psycho-Social Lifestyle Profile. A cross-sectional non-randomized design will be used to determine if PMPs can be feasibility assessed.

Table 1. Summary of the measurements, outcomes, and instruments to feasibly assess PMPs.

Primary outcome	Instrument	Measurement
Feasibility	Personalized questionnaire	Process metrics
		Resource metrics
		Management metrics
		Scientific metrics
	UEQ	Operational feasibility
**Profiling related outcome**	**Instrument**	**Measurement**
Sociodemographics	Questionnaire	Age – Gender – Body mass index
		Socio-economic status
Headache characteristics	Headache-diary	Intensity – Frequency – Duration – Medication intake
Pain Profile	Pressure cuff	Conditioned Pain Modulation (conditioning stimulus)
	Algometer	Conditioned Pain Modulation (test stimulus)
		Widespread hyperalgesia
	Pinprick stimulator	Temporal Summation
Psycho-Social Lifestyle Profile	DASS-21	Depression, Anxiety, Stress
	PSQI	Sleep Quality
	HIT-6	Quality of Life

UEQ = User Experience Questionnaire; DASS-21 = Depression, Anxiety, Stress Scale; PSQI = Pittsburgh Sleep Quality Index; HIT-6 = Headache Impact Test.

2.2 Study population and setting

Participants with CeH will be screened and recruited via the neurological staff of the headache department of the AZ Vesalius hospitals (Tongeren and Bilzen, Belgium).

A detailed summary of the study population's inclusion and exclusion criteria is provided in Supplemental Table S.1. A four-week headache-diary will be distributed among the enrolled

For peer review only - http://bmjopen.bmj.com/site/about/guidelines.xhtml

participants to collect information on the following headache characteristics: intensity, frequency, duration, and medication intake. Eligible participants will need to read and sign the informed consent (collected by the principal researcher) before officially being enrolled. All test procedures involving human participants will be in accordance with the ethical standards of the institutional research committees and with the 1964 Helsinki Declaration and its later amendments. The study is submitted to the Ethical Committee Research UZ / KU Leuven (reference S67191). Set-up will be at the Leuven University (Leuven, Belgium) and private practice of the principal researcher (Riemst, Belgium).

2.3 Patient and public Involvement

People with CeH will be involved in the feasibility study. This study aims to gather information on arguments to participate or decline to participate, reason(s) for premature ending, appropriateness of the measurements, and barriers, adverse events, or burden experienced by people with CeH. The information will be used to adapt the protocol. Feedback on possible adaptations will be provided.

2.4 Experimental protocol

A PMP, consisting of a Psycho-Social Lifestyle Profile and Pain Profile, will be composed (Figure 1). Three questionnaire will be used to estimate the Psycho-Social Lifestyle Profile. The Pain Profile will be interpreted based on three parameters (TSP, CPM, widespread hyperalgesia).

[Figure 1]

Assessment will be in a headache-free phase (= score of 0 on the Numeric Pain Rating Scale). Participants will be informed on possible adverse events, and asked to abstain from vigorous physical activity, taking analgesics, and caffeine-containing beverages 24 hours prior to the measurements. Prophylactic treatment(s) remain unchanged (1). Adverse events will be questioned after finalizing the test-procedures, and again after 24 hours. Measurements will be performed by the principal researcher (manual therapist, > 10 years clinical experience, PhD Physiotherapy & Rehabilitation Sciences) in a chronological order (Figures 2-4). The entire procedure is estimated to amount to 4 hours. An overview of the time schedule for the feasibility study is provide in Supplemental Figure S.2.

2.4.1 Psycho-Social Lifestyle Profile - Questionnaires

The Pittsburgh Sleep Quality Index (PSQI), Depression, Anxiety, Stress Scale (DASS-21), and Headache Impact Test (HIT-6) will be completed in a quiet room prior to the CPM, TSP, and Pressure Pain

For peer review only - http://bmjopen.bmj.com/site/about/guidelines.xhtml

Threshold measurements. Thereafter the User Experience Questionnaire will be filled in. The principal researcher will be available in case of ambiguity about the questions/statements.

2.4.2 Pain Profile Temporal Summation of Pain

Figure 2 provides a summary of the TSP protocol.

[Figure 2]

2.4.3 Conditioned Pain Modulation

Figure 3 provides a summary of the CPM protocol which will be executed 15 minutes after the TSP measurements.

[Figure 3]

2.4.4 Widespread hyperalgesia

Figure 4 provides a summary of the protocol to evaluate widespread hyperalgesia which will be executed 15 minutes after the CPM measurements (Supplement 3).

[Figure 4]

2.5 Measurements, outcomes, instruments, and procedure

2.5.1 Primary outcome – Feasibility

Feasibility of the protocol will be assessed using adapted feasibility metrics. These metrics include: process, resource, management, and scientific metrics (36,37). Details on the primary outcomes are summarized in Supplemental Table S.4.

For peer review only - http://bmjopen.bmj.com/site/about/guidelines.xhtml

2.5.2 Profiling-related outcome – Psycho-Social Lifestyle Profile

Participants will be categorised in Psycho-Social Lifestyle Profile based on the number of deviating psychosocial-lifestyle factors deducted from scores on the PSQI, DASS-21, and HIT-6. Individual scores will be compared to normative data (17). Scores indicating at least: moderate depression, anxiety, stress (DASS-21), headache has a significant impact on daily life (HIT-6), and/or poor sleep quality (PSQI) will be considered as deviating.

2.5.2.1 Psycho-Social Lifestyle factors

Sleep quality will be assessed via the Dutch PSQI, a standardized, valid, and reliable self-reported one-month recall questionnaire (39,40). The index differentiates poor from good sleepers by measuring: subjective sleep quality, sleep latency, sleep duration, habitual sleep efficiency, sleep disturbances, use of sleeping medication, and daytime dysfunction. Scores on each of these components vary from 0 ('No problem') to 3 ('Serious problem'). A maximum score exceeding 5/21 indicates poor sleep quality (41,42). See Mollayeva et al. (2016) for information on the psychometric properties (39).

The degree of *depression, anxiety, and stress* will be estimated by the Dutch DASS-21 (43,44). The DASS-21 is a self-reported, one-week recall questionnaire. Each of the three sub-scales contain seven items. The depression subscales assess dysphoria, hopelessness, devaluation of life, self-deprecation, lack of interest, anhedonia, and inertia. The anxiety subscale estimates autonomic arousal, skeletal muscle effects, situational anxiety, and subjective experience of anxious affect. The stress subscale evaluates difficulty in relaxing, nervous arousal, being easily upset, and impatience. Items are scored on a Likert-scale (0 = 'Did not apply to me at all', and 3 = 'Applied to me very much or most of the time'). Scores of 14, 10, and 19 indicate at least moderate depression, anxiety, and stress, respectively. See Lovibond et al. (1995) for information on the psychometric properties (45).

Impact *of headache on quality of life* will be estimated by the Dutch HIT-6 (46,47). The HIT-6 evaluates the impact of headache on daily activities: the ability to function at work, school, home, and in social situations. Items are scored 6, 8, 10, 11, and 13 (6 = 'Never', 8 = 'Rarely', 10 = 'Sometimes', 11 = 'Very often', and 13 = 'Always'). Scores exceeding 56 indicate headache has a significant impact on daily life (47,48). See Martin et al. (2004) and Kosinski et al. (2003) for information on the psychometric properties (47,49).

For peer review only - http://bmjopen.bmj.com/site/about/guidelines.xhtml

2.5.3 Profiling-related outcome – Pain Profile

Participants will be categorised in four Pain Profiles based on responses to the TSP and CPM (Figures 1-3):

P-P I: Pronociceptive (increased TSP/impaired CPM)

P-P II: Pronociceptive (normal TSP/impaired CPM)

P-P III: Pronociceptive (increased TSP/normal CPM)

P-P IV: Antinociceptive (normal TSP/normal CPM)

Each participant with CeH will be categorised into a Pain Profile per measurement location (TSP at the hand and face, CPM at the left and right suboccipital muscles) (50,51). Additional presence of widespread pain will be evaluated (Figure 4). The paragraphs below contain information on the definitions of increased/decreased TSP, impaired/normal CPM, and presence of widespread hyperalgesia.

2.5.3.1 Pinprick stimulus: Temporal summation of Pain (TSP)

The pinprick evoked Wind-Up Ratio (WUR) (Pinprick stimulator, MRC Systems GmbH - Medizintechnische Systeme) is suggested to evaluate TSP. Participants will be positioned in sitting. TSP at the bilateral (i.e. painful and pain-free sides) ophthalmic region (V1), and extra-segmentally on the thenar eminence of the dominant hand will be evaluated via pinprick evoked WUR (MRC Systems GmbH-Medizintechnische Systeme, Heidelberg, Germany) (50,52-54). WUR represents the quotient of pain intensity evoked by one single pinprick stimulus divided by the average pain evoked by 10 repetitive pinprick stimuli (52-54). We additionally propose to apply the SumSquare method as described by Allison et al. (2021) to quantifying TSP (55). The WUR is reported to lack sensitivity to detect differences in the magnitude of sensitisation. Most studies report little variability (ratio 1.6-1.7) in WUR measurements both in normal and hyperalgesic states (55).

For the measurements at the face and hand constant pinpricks of 128 mN and 256mN, respectively will be used (53). First, a single pinprick stimulus will be applied, followed by a series of 10 pinprick stimuli with a frequency of 1 Hz within an area of 1 cm^2 (51,53). The interval between the single, and repetitive stimuli will be 1 minute, or until all aftersensations have resolved. Participants will be asked to rate the pain of a single pinprick stimulus, and the average pain at the end of the series of 10 pinpricks using the 0-10 Numeric Pain Rating Scale (0 = no pain, 10 = the worst pain imaginable). The test order will be standardized as: dominant hand – left side face – right side face. This procedure will be repeated three times at each site, and three measurements per site will be averaged (Figure 2). We refer to the normative values provided by the German Research Network on Neuropathic Pain to determine normal or increased TSP at the face and hand (52). Test-retest reliability for pinprick induced TSP is moderate-to-good in healthy participants (ICC 0.51 – 0.61) (56).

For peer review only - http://bmjopen.bmj.com/site/about/guidelines.xhtml

2.5.3.2 Test stimulus: Mechanical pressure pain thresholds and tolerance

Measurements will be performed 15 minutes after the TSP measurements. Pressure pain threshold (PPT) and pressure pain tolerance (PPTo) (kPa/cm²) will be measured at the left and right suboccipital muscles using an electronic pressure algometer (Somedic AB, Stockholm, Sweden) (57). Pressure will be perpendicularly applied directly on the suboccipital muscles, starting at 0 to maximal 1000 kPa, using a 1 cm² probe with a slope of 30 kPa/s (17,58). Participants will be instructed to push the stop-button when the sensation of pressure first elicits pain (PPT), and when the maximum amount of pressure that could be tolerated is reached (PPTo). A trial will be performed once on the left shin (tibialis anterior muscle belly, approximately 2.5 cm lateral and 5 cm inferior to the tibial tubercle) before measuring the suboccipital muscles (59). Intrarater reliability of tibialis anterior PPT-measurements are good-to-excellent in people with CeH (ICC 0.82-0.92) (60).

Two repeated measurements at baseline, and two measurements parallel with the conditioning stimulus will be performed with (Figure 3). Averages of the extracted parameters will be used for further analysis (61). Test-retest reliability of PPT-measurements at the suboccipital muscles is good to excellent (ICC 0.84 – 0.93) (62). Intra-rater reliability of such PPT-measurements are moderate-to-good in people with CeH (ICC 0.69 - 0.87) (17,63).

2.5.3.3 Conditioning stimulus: Cuff pressure algometry

Cuff pressure tolerance (kPa) will be measured at the non-dominant lower leg. A cuff (Nocitech, Aalborg University, Denmark) will be placed around the bare non-dominant calf muscle, mounted with 8-cm distance between its upper border and the tibial tuberosity (64,65). Upper and lower borders of the cuff will be marked on the skin to ensure the cuff does not move between stimulations. Pressure tolerance will be determined through the maximal amount of pressure that can be tolerated. Intensity of the cuff pressure for the conditioning stimulus will be predefined as 70% of the pressure tolerance on the non-dominant leg (64,65). Computer-controlled cuff algometry (LabBench® CPAR+ instrument) shows an excellent intra-rater reliability (ICC 0.89) in healthy subjects (67,68). Test-retest reliability is good (ICCs 0.74 – 0.87) in healthy subjects (64).

2.5.3.4 Conditioned pain modulation (CPM)

CPM will be assessed 3 minutes after determining cuff pressure tolerance. The pressure (conditioning stimulus) will be kept constant throughout the CPM protocol (65,66). Five seconds after inflation of the cuff, PPT and PPTo will be reassessed as described above. Participants will be informed that the conditioning stimulus will be moderately painful, but that they should focus their attention on the test stimulus. The procedure described above will be sequentially and twice executed, i.e. once with the PPT-measurement at the left suboccipital muscles, and once with the PPT-measurement at the right

For peer review only - http://bmjopen.bmj.com/site/about/guidelines.xhtml

suboccipital muscles as test stimulus. A 15-minute-interval will be provided between the CPM-measurements since CPM has a short lasting (< 15 minutes) hypoalgesic effect (Figure 3) (69-72).

At the moment, there is no consensus on a normal CPM effect. We propose a method described by Vaegter et al. (2016) to categorize the CPM response as normal or impaired based on the within-subject coefficient of variation (= within-subject SD/within-subject mean) in PPT. Participants will be profiled as having an impaired CPM if the CPM response is less than or equal to the normal within-subject coefficient of variation in PPT between 2 repeated assessments, and as having normal CPM if the CPM response is greater than the normal variation plus the upper limit of the 95% confidence interval (61).

2.5.3.5 Widespread hyperalgesia

Widespread hyperalgesia indicates facilitated central pain mechanisms (73). PPT and CPM measurements will be used to estimate widespread hyperalgesia.

PPTs at both symptomatic (bilateral suboccipital muscles) and distant pain-free (bilateral tibialis anterior muscles) areas will be used to determine widespread hyperalgesia. PPTs lower than the 95% confidence interval lower border bound of the normative PPTs will be considered decreased (= deviating) (17). PPTs at the suboccipitals will not be repeated since data are already collected within the context of CPM (cfr. paragraph 2.5.3.4). PPTs of the bilateral tibialis anterior muscle will be determined as described above (cfr. paragraph 2.5.3.2). The test order will be standardized as: left tibialis anterior – right tibialis anterior muscle. This procedure will be repeated two times, and measurements per site will be averaged. Averages of the extracted parameters will be used for further analysis (61). An extra-segmental *CPM measurement* will be performed as second proxy to estimate widespread hyperalgesia (Supplement 3).

2.6 Data Management

Data concerning feasibility, CPM, TSP, and widespread hyperalgesia will be electronically collected (.xlsx). Data gathered from the paper questionnaires (PSQI, DASS-21, HIT-6) will be transferred to excel files (.xlsx). Data will be protected by pseudonymising them, restricting access, and access only via the multifactor-authentication (KU Leuven Authenticator app). The principal researcher will complete the pseudonymising process and GDPR-questionnaire according to the KU Leuven policy. Storing is foreseen in a folder structure (OneDrive, Microsoft 365 Apps for enterprise) to secure: availability of data, confidentiality, and sharing data (https://icts.kuleuven.be/storagewijzer/nl). Finished data (i.e. the dataset) will be stored at the KU Leuven Research Data Repository (https://www.kuleuven.be/rdm/en/rdr/rdr). This platform enables archiving (at the end of the

For peer review only - http://bmjopen.bmj.com/site/about/guidelines.xhtml

project), uploading, describing, and sharing research data in a legal and controlled manner. Exclusively the principal researcher and principal investigator will have access to all data, other team members will only obtain restricted access. The latter implies that data are anonymous for these team members. All data will be kept for 10 years, conform the KU Leuven policy. Supportive arguments to keep personal data for 10 years include: verification of results and future research. Final versions of datasets will be preserved for the long-term on internal KU Leuven data storage facilities.

2.7 Statistics

Primary analyses will report results relating to outcomes on feasibility. Descriptive statistics and content analysis will be used to assess feasibility of the protocol. The feasibility study will provide useful information with regard to planning, testing study procedures (e.g. estimation of the recruitment rate, plausibility of multicentre collaborations, etc.), and investigating outcomes to support adequate sample size calculation (74). Data from the CMP and TSP measurements will be used to estimate post hoc sample size (G*Power 3.1.9.4, Kiel Germany) for a larger trial.

Individual PMPs of at least 12 participants with CeH will be mapped based on the methods outlined in sections 2.5.2 and 2.5.3. Secondary analyses will involve an analysis of proportions (%) of the different Psycho-Social Lifestyle Profiles and Pain Profiles. Currently no data are available in people with CeH, therefore a pragmatic approach is proposed based on the work of Julious (2005) who recommended 12 participants as the minimum for pilot studies (75). Justifications for this sample size are based on rationale about feasibility and precision about the mean and variance (75). Both primary and secondary analyses will be used to decide whether the feasibility study should be expanded to a full scale study. The latter implies registering of the trial in accordance the World Health Organisation Trial Registration Data Set (V 1.3.1).

3. DISCUSSION

3.1 Relevance of the study

"Maybe not all therapeutic interventions are appropriate for all people with CeH" (12). With this statement Fernández-de-Las-Peñas et al. (2014) drew attention to an important problem in the management of CeH. Others have hypothesized that not all people with CeH can be managed successfully by only targeting the upper-cervical spine (12,13,15). Chua et al. (2011) advised that the management of CeH should not exclusively focus on the cervical nociceptive source, but also on central nervous system sensitization spreading to the level of the trigeminal spinal nucleus (76).

Findings of previous work of our group confirmed this by identifying secondary and tertiary hyperalgesia (i.e. increased pain sensitivity in undamaged tissue away from, and contralateral of the nociceptive source). Meaning that both bilateral cephalic and extra-cephalic PPTs were lower in people

For peer review only - http://bmjopen.bmj.com/site/about/guidelines.xhtml

with CeH compared to healthy asymptomatic controls (17). However, these findings do not imply that peripheral nociceptive sources should be ignored. Such sources could initiate, maintain, and modulate facilitated central pain processes. Therefore, potential nociceptive sources alongside psychological, behavioural, and social components should also be assessed (i.e. interplay of bottom-up and top-down mechanisms) (77).

Although CPM-testing was already applied to people experiencing various types of headache such as chronic migraine, tension-type headache, and post-traumatic headache (18,78-80), it was only once administered to people with chronic CeH (76). These people with chronic CeH experienced moderate pain (numeric pain rating scale 6.5 ± 1.8) during CPM-testing. We propose to analyse endogenous pain modulation during a headache-free phase. Having a less efficient CPM when being headache-free, suggests that upon a pain-generating event, the person is at a higher risk to develop pain than people showing an efficient CPM at baseline (81).

3.2 The Protocol

We propose a protocol to explore feasibility to assess PMPs in people with CeH. Inter-individual variability in endogenous pain modulation is expected in these people due to their heterogeneous profile (17,60,82,83). Reduced endogenous pain modulation might be expected in people with CeH presenting with modifiable lifestyle and/or psychosocial risk factors for chronification. This hypothesis is supported by PPTs at the suboccipital muscles being influenced by the level of physical activity, stress, quality of life, and screen-time in people with CeH (17). Therefore, we will map the multidimensional PMP (i.e. Psycho-Social Lifestyle Profile and Pain Profile) of each participant. Knowledge of a person's profile might support an individual management program as opposed to the traditional one-size-fits-all approach (16).

We opted to use a dynamic protocol to assess the Pain Profile of people with CeH rather than relying exclusively on questionnaires and/or static measurements. Caution is indicated when screening questionnaires (e.g. central sensitization inventory) are used to identify human assumed central sensitization. These questionnaires are not associated with widespread pain sensitivity, and show a stronger association with psychological measures than with signs of facilitated central pain mechanisms (84).

3.3 Feasibility

Feasibility of the protocol is anticipated since it is designed according to the latest literature in the domain of TSP and CMP measurements (24,52,53,56,66,69,70,85,86). Collaboration with the AZ Vesalius hospitals (Tongeren and Bilzen, Belgium) is foreseen to obtain the required sample size of 12 people with CeH (17,82,83).

For peer review only - http://bmjopen.bmj.com/site/about/guidelines.xhtml

A mitigation plan is composed to anticipate potential adverse events related to the testing procedure such as light headache, muscle soreness or sensitivity caused by mechanical pressure on cutaneous, muscular, nervous or arterial tissue. This plan includes general precautions such as informing participants on possible transient adverse effects, applying a strict protocol, using a wide cuff at the least uncomfortable test-location (i.e. calf muscles), and asking to abstain from vigorous physical activity at least 24 hours prior to the measurement. Potential adverse effects are expected to resolve within a 10 minutes recovery period (87). This feasibility study will provide useful information with regard to tolerability, planning, testing study procedures (e.g. estimation of the recruitment rate, plausibility of multicentre collaborations), and investigating outcomes to support adequate sample size calculation (74). Further, such study is designed to ensure that a possible future larger intervention study is achievable, rigorous, and economically justifiable to avoid waste of resources (74). In the current context of a feasibility study, a Data Monitoring Committee will not be involved. Adverse events are expected to be minor and transient in nature, and no long-term follow-up or interventions are applied.

Further, measurements will be conducted with a respected interval (15 minutes after CPM) to limit carry-over of the responses (71,72).

3.4 Limitations and suggestions

We acknowledge that assessing facilitated central pain mechanisms clinically is challenging for several reasons such as the absence of biomarkers to support its diagnosis. Although quantitative sensory testing is a promising surrogate measure to identify such facilitated mechanisms, it only evaluates evoked responses rather than spontaneous pain (84). Results should be interpreted within that context.

More research is needed into relevant modifiable psychosocial and lifestyle factors concerning sensitization of pain in CeH (88). Sedentary-time, anxiety, sleep quality, physical activity, and stress were already reported to be associated with signs of central sensitization in some people with CeH (17). Additionally, inter-individual differences explain a large proportion of CPM-variance (89). This finding forces researchers to look at the CeH-population as more heterogeneous (17,60). We propose a feasibility study which includes only 12 participants with CeH, which is the minimum recommended for pilot studies (75).

Further, WUR is reported to lack sensitivity to detect differences in the magnitude of sensitisation. Most studies report little variability (ratio 1.6-1.7) in WUR measures both in normal and hyperalgesic states. Therefore we additionally propose to apply the SumSquare method as described by Allison et al. (2021) (55). Finally, the potential future full scale study should analyse endogenous pain modulation within a repeated-measures design to detect variance (i.e. inter-individual differences) (89).

For peer review only - http://bmjopen.bmj.com/site/about/guidelines.xhtml

For peer review only

4. CONCLUSION

A protocol for a feasibility study to assess PMPs among people with CeH is described. Conducting a feasibility study to evaluate this protocol is warranted before developing a stepwise path of care based on the dominant pain mechanism in people with CeH.

5. FUNDING

AS was funded in whole, or in part, by the Wellcome Trust (222101/Z/20/Z). For the purpose of open access, the author has applied a CC BY public copyright license to any Author Accepted Manuscript version arising from this submission.

TGN is a part of the Center for Neuroplasticity and Pain (CNAP) supported by the Danish National Research Foundation (DNRF121).

SM is supported by the Leuven University (PDMt1/22/016). The sponsor has no role in composing the study design, collection, management, analysis, and interpretation of data, writing of the report, or the decision to submit the report for publication.

6. DECLARATION OF INTEREST

The authors declare no conflict of interest.

7. AUTHOR CONTRIBUTIONS

SM drafted the article. AS, TGN, MG, and WD revised the article critically for essential intellectual and methodological content. All authors approved the version to be published.

8. REFERENCES

1. Headache Classification Committee of the International Headache Society (IHS) The International Classification of Headache Disorders, 3rd edition. Cephalalgia 2018;38(1):1-211.
2. Fredriksen TA, Antonaci F, Sjaastad O. Cervicogenic headache: too important to be left un-diagnosed. J Headache Pain 2015;16:6.
3. Kristoffersen ES, Stavem K, Lundqvist C, et al. Impact of chronic headache on workdays, unemployment and disutility in the general population. Journal of Epidemiology and Community Health 2019;73(4):360-7.
4. Raja SN, Carr DB, Cohen M, et al. The revised International Association for the Study of Pain definition of pain: concepts, challenges, and compromises. Pain 2020;161(9):1976-82.
5. Wijma AJ, van Wilgen CP, Meeus M, et al. Clinical biopsychosocial physiotherapy assessment of patients with chronic pain: The first step in pain neuroscience education. Physiother Theory Pract 2016;32(5):368-84.
6. De Pauw R, Dewitte V, de Hertogh W, et al. Consensus among musculoskeletal experts for the management of patients with headache by physiotherapists? A delphi study. Musculoskelet Sci Pract 2021;52:102325.

For peer review only - http://bmjopen.bmj.com/site/about/guidelines.xhtml

7. Varatharajan S, Ferguson B, Chrobak K, et al. Are non-invasive interventions effective for the management of headaches associated with neck pain? An update of the Bone and Joint Decade Task Force on Neck Pain and Its Associated Disorders by the Ontario Protocol for Traffic Injury Management (OPTIMa) Collaboration. Eur Spine J 2016;25(7):1971-99.
8. Moore CS, Sibbritt DW, Adams J. A critical review of manual therapy use for headache disorders: prevalence, profiles, motivations, communication and self-reported effectiveness. BMC Neurol. 2017;17(1):61.
9. Gross AR, Paquin JP, Dupont G, et al. Exercises for mechanical neck disorders: A Cochrane review update. Man Ther 2016;24:25-45.
10. Fernandez M, Moore C, Tan J, et al. Spinal manipulation for the management of cervicogenic headache: A systematic review and meta-analysis. Eur J Pain 2020;24(9):1687-702.
11. Coelho M, Ela N, Garvin A, et al. The effectiveness of manipulation and mobilization on pain and disability in individuals with cervicogenic and tension-type headaches: a systematic review and meta-analysis. Phys Ther Rev 2019;24:29-43.
12. Fernández-de-las-Peñas C, Cuadrado ML. Therapeutic options for cervicogenic headache. Expert Rev Neurother 2014;14(1):39-49.
13. Fernández-de-Las-Peñas C, Florencio LL, Plaza-Manzano G, et al. Clinical Reasoning Behind Non-Pharmacological Interventions for the Management of Headaches: A Narrative Literature Review. Int J Environ Res Public Health 2020;17(11):4126.
14. Woolf CJ. Central sensitization: implications for the diagnosis and treatment of pain. Pain 2011;152(3 Suppl):S2-s15.
15. Nijs J, George SZ, Clauw DJ, et al. Central sensitisation in chronic pain conditions: latest discoveries and their potential for precision medicine. The Lancet Rheumatology 2021;3(5):e383-e92.
16. Yarnitsky D, Granot M, Granovsky Y. Pain modulation profile and pain therapy: between pro- and antinociception. Pain 2014;155(4):663-5.
17. Mingels S, Dankaerts W, van Etten L, et al. Exploring multidimensional characteristics in cervicogenic headache: Relations between pain processing, lifestyle, and psychosocial factors. Brain Behav 2021;11(10):e2339.
18. Staud R. Abnormal endogenous pain modulation is a shared characteristic of many chronic pain conditions. Expert Rev Neurother 2012;12(5):577-85.
19. Yarnitsky D, Arendt-Nielsen L, Bouhassira D, et al. Recommendations on terminology and practice of psychophysical DNIC testing. Eur J Pain 2010;14(4):339.
20. Eide PK. Wind-up and the NMDA receptor complex from a clinical perspective. Eur J Pain 2000;4(1):5-15.
21. Price DD, Hayes RL, Ruda M, et al. Neural representation of cutaneous aftersensations by spinothalamic tract neurons. Fed Proc 1978;37(9):2237-9.
22. Arendt-Nielsen L, Morlion B, Perrot S, et al. Assessment and manifestation of central sensitisation across different chronic pain conditions. Eur J Pain 2018;22(2):216-41.
23. Daenen L, Nijs J, Cras P, et al. Changes in Pain Modulation Occur Soon After Whiplash Trauma but are not Related to Altered Perception of Distorted Visual Feedback. Pain Pract 2014;14(7):588-98.
24. Kennedy DL, Kemp HI, Ridout D, et al. Reliability of conditioned pain modulation: a systematic review. Pain 2016;157(11):2410-9.
25. Staud R, Vierck CJ, Cannon RL, et al. Abnormal sensitization and temporal summation of second pain (wind-up) in patients with fibromyalgia syndrome. Pain 2001;91(1-2):165-75.
26. Lacey RJ, Campbell P, Lewis M, et al. The Impact of Inadequate Health Literacy in a Population with Musculoskeletal Pain. Health Lit Res Pract 2018;2(4):e215-e20.
27. Liebert A, Rebbeck T, Elias S, et al. Musculoskeletal physiotherapists' perceptions of non-responsiveness to treatment for cervicogenic headache. Physiother Theory Pract 2013;29(8):616-29.
28. Racine M, Tousignant-Laflamme Y, Kloda LA, et al. A systematic literature review of 10 years of research on sex/gender and pain perception - part 2: do biopsychosocial factors alter pain sensitivity differently in women and men? Pain 2012;153(3):619-35.
29. Yarnitsky D. Role of endogenous pain modulation in chronic pain mechanisms and treatment. Pain 2015;156.
30. Dhondt E, Danneels L, Van Oosterwijck S, et al. The influence of physical activity on the nociceptive flexion reflex in healthy people. Eur J Pain 2021;25(4):774-89.
31. De Felice M, Ossipov MH. Cortical and subcortical modulation of pain. Pain Management 2016;6(2):111-–20.
32. Bobinski F, Ferreira TAA, Córdova MM, et al. Role of brainstem serotonin in analgesia produced by low-intensity exercise on neuropathic pain after sciatic nerve injury in mice. Pain 2015;156(12):2595-606.
33. Sluka KA, O'Donnell JM, Danielson J, et al. Regular physical activity prevents development of chronic pain and activation of central neurons. J Appl Physiol 2013;114(6):725-33.
34. Stagg NJ, Mata HP, Ibrahim MM, et al. Regular exercise reverses sensory hypersensitivity in a rat neuropathic pain model: role of endogenous opioids. Anesthesiology 2011;114(4):940-8.
35. Staffe AT, Bech MW, Clemmensen SLK, et al. Total sleep deprivation increases pain sensitivity, impairs conditioned pain modulation and facilitates temporal summation of pain in healthy participants. PLoS One 2019;14(12):e0225849.
36. Learmonth YC, Motl RW. Important considerations for feasibility studies in physical activity research involving persons with multiple sclerosis: a scoping systematic review and case study. Pilot Feasibility Stud 2018;4:1.
37. Thorpe N, Harniess P, Main E, et al. Feasibility, safety and acceptability of select outcome measures in a physiotherapy study protocol for boys with haemophilia. Pilot Feasibility Stud 2021;7(1):105.

For peer review only - http://bmjopen.bmj.com/site/about/guidelines.xhtml

38. Laugwitz B. Construction and Evaluation of a User Experience Questionnaire. HCI and Usability for Education and Work. Heidelberg: Springer Berlin Heidelberg 2008
39. Mollayeva T, Thurairajah P, Burton K, et al. The Pittsburgh sleep quality index as a screening tool for sleep dysfunction in clinical and non-clinical samples: A systematic review and meta-analysis. Sleep Med Rev 2016;25:52-73.
40. Marinus J, Visser M, van Hilten JJ, et al. Assessment of sleep and sleepiness in Parkinson disease. Sleep 2003;26(8):1049-54.
41. Buysse DJ, Reynolds CF, Monk TH, et al. The Pittsburgh Sleep Quality Index: a new instrument for psychiatric practice and research. Psychiatry Res 1989;28(2):193-213.
42. Smyth CA. Evaluating sleep quality in older adults: the Pittsburgh Sleep Quality Index can be used to detect sleep disturbances or deficits. Am J Nurs 2008;108(5):42-50; quiz -1.
43. Parkitny L, McAuley JH, Walton D, et al. Rasch analysis supports the use of the depression, anxiety, and stress scales to measure mood in groups but not in individuals with chronic low back pain. J Clin Epidemiol 2012;65(2):189-98.
44. de Beurs E, Van Dyck R, Marquenie LA, et al. De DASS: Een vragenlijst voor het meten van depressie, angst en stress. [The DASS: A questionnaire for the measurement of depression, anxiety, and stress.]. Gedragstherapie 2001;34(1):35-53.
45. Lovibond PF, Lovibond SH. The structure of negative emotional states: comparison of the Depression Anxiety Stress Scales (DASS) with the Beck Depression and Anxiety Inventories. Behav Res Ther 1995;33(3):335-43.
46. Buse DC, Sollars CM, Steiner TJ, et al. Why HURT? A review of clinical instruments for headache management. Curr Pain Headache Rep 2012;16(3):237-54.
47. Martin M, Blaisdell B, Kwong JW, et al. The Short-Form Headache Impact Test (HIT-6) was psychometrically equivalent in nine languages. J Clin Epidemiol 2004;57(12):1271-8.
48. Kawata AK, Coeytaux RR, Devellis RF, et al. Psychometric properties of the HIT-6 among patients in a headache-specialty practice. Headache 2005;45(6):638-43.
49. Kosinski M, Bayliss MS, Bjorner JB, et al. A six-item short-form survey for measuring headache impact: the HIT-6. Qual Life Res 2003;12(8):963-74.
50. Moana-Filho EJ, Herrero Babiloni A. Endogenous pain modulation in chronic temporomandibular disorders: Derivation of pain modulation profiles and assessment of its relationship with clinical characteristics. J Oral Rehabil 2019;46(3):219-32.
51. Gil-Martínez A, Grande-Alonso M, La Touche R, et al. Psychosocial and Somatosensory Factors in Women with Chronic Migraine and Painful Temporomandibular Disorders. Pain Res Manag 2016;2016:3945673.
52. Rolke R, Magerl W, Campbell KA, et al. Quantitative sensory testing: a comprehensive protocol for clinical trials. Eur J Pain 2006;10(1):77-88.
53. Rolke R, Baron R, Maier C, et al. Quantitative sensory testing in the German Research Network on Neuropathic Pain (DFNS): standardized protocol and reference values. Pain 2006;123(3):231-43.
54. Baeumler PI, Conzen P, Irnich D. High Temporal Summation of Pain Predicts Immediate Analgesic Effect of Acupuncture in Chronic Pain Patients-A Prospective Cohort Study. Front Neurosci 2019;13:498.
55. Allison C, Korey L, John ZS. A novel computational technique for the quantification of temporal summation in healthy individuals. Musculoskelet Sci Pract 2021;54:102400.
56. Marcuzzi A, Wrigley PJ, Dean CM, et al. The long-term reliability of static and dynamic quantitative sensory testing in healthy individuals. Pain 2017;158(7):1217-23.
57. Bogduk N. The neck and headaches. Neurol Clin 2014;32(2):471-87.
58. Alburquerque-Sendín F, Madeleine P, Fernández-de-Las-Peñas C, et al. Spotlight on topographical pressure pain sensitivity maps: a review. J Pain Res 2018;11:215-25.
59. Walton DM, Macdermid JC, Nielson W, et al. Reliability, standard error, and minimum detectable change of clinical pressure pain threshold testing in people with and without acute neck pain. J Orthop Sports Phys Ther 2011;41(9):644-50.
60. Mingels S, Dankaerts W, Bruckers L, et al. Inter-individual variability in mechanical pain sensation in patients with cervicogenic headache: an explorative study. Sci Rep 2022;12(1):20635.
61. Vaegter HB, Graven-Nielsen T. Pain modulatory phenotypes differentiate subgroups with different clinical and experimental pain sensitivity. Pain 2016;157(7):1480-8.
62. Ylinen J, Nykänen M, Kautiainen H, et al. Evaluation of repeatability of pressure algometry on the neck muscles for clinical use. Man Ther 2007;12(2):192-7.
63. Zicarelli CAM, Santos JPM, Poli-Frederico RC, et al. Reliability of pressure pain threshold to discriminate individuals with neck and low back pain. J Back Musculoskelet Rehabil 2021;34(3):363-70.
64. Cummins TM, Kucharczyk MM, Graven-Nielsen T, et al. Activation of the descending pain modulatory system using cuff pressure algometry: Back translation from man to rat. Eur J Pain 2020;24(7):1330-8.
65. Graven-Nielsen T, Vaegter HB, Finocchietti S, et al. Assessment of musculoskeletal pain sensitivity and temporal summation by cuff pressure algometry: a reliability study. Pain 2015;156(11):2193-202.
66. Hoegh M, Petersen KK, Graven-Nielsen T. Effects of repeated conditioning pain modulation in healthy volunteers. Eur J Pain. 2018;22(10):1833-43.
67. Inventors'Way ApS. Cuff Pressure Algometry [online]. 2023. https://nocitech.com (accessed April 15th 2023).
68. Kvistgaard Olsen J, Fener DK, Wæhrens EE, et al. Reliability of Pain Measurements Using Computerized Cuff Algometry: A DoloCuff Reliability and Agreement Study. Pain Pract 2017;17(6):708-17.
69. Lindskou TA, Christensen SW, Graven-Nielsen T. Cuff Algometry for Estimation of Hyperalgesia and Pain Summation. Pain Med 2017;18(3):468-76.

For peer review only - http://bmjopen.bmj.com/site/about/guidelines.xhtml

70. Graven-Nielsen T, Izumi M, Petersen KK, et al. User-independent assessment of conditioning pain modulation by cuff pressure algometry. Eur J Pain 2017;21(3):552-61.
71. Vaegter HB, Handberg G, Graven-Nielsen T. Similarities between exercise-induced hypoalgesia and conditioned pain modulation in humans. Pain 2014;155(1):158-67.
72. Vaegter HB, Handberg G, Jørgensen MN, et al. Aerobic exercise and cold pressor test induce hypoalgesia in active and inactive men and women. Pain Med 2015;16(5):923-33.
73. Graven-Nielsen T, Arendt-Nielsen L. Assessment of mechanisms in localized and widespread musculoskeletal pain. Nat Rev Rheumatol 2010;6(10):599-606.
74. Thabane L, Ma J, Chu R, et al. A tutorial on pilot studies: the what, why and how. BMC Med Res Methodol 2010;10:1.
75. Julious SA. Sample size of 12 per group rule of thumb for a pilot study. Pharmaceutical Statistics: The Journal of Applied Statistics in the Pharmaceutical Industry 2005;4(4):287-91.
76. Chua NH, van Suijlekom HA, Vissers KC, et al. Differences in sensory processing between chronic cervical zygapophysial joint pain patients with and without cervicogenic headache. Cephalalgia 2011;31(8):953-63.
77. van Griensven H, Schmid A, Trendafilova T, et al. Central Sensitization in Musculoskeletal Pain: Lost in Translation? J Orthop Sports Phys Ther 2020;50(11):592-6.
78. Nahman-Averbuch H, Granovsky Y, Coghill RC, et al. Waning of "conditioned pain modulation": a novel expression of subtle pronociception in migraine. Headache 2013;53(7):1104-15.
79. Defrin R, Riabinin M, Feingold Y, et al. Deficient pain modulatory systems in patients with mild traumatic brain and chronic post-traumatic headache: implications for its mechanism. J Neurotrauma 2015;32(1):28-37.
80. Exposto FG, Bendixen KH, Ernberg M, et al. Assessment of Pain Modulatory and Somatosensory Profiles in Chronic Tension-Type Headache Patients. Pain Med 2021;22(10):2356-65.
81. Yarnitsky D. Role of endogenous pain modulation in chronic pain mechanisms and treatment. Pain 2015;156:S24-S31.
82. Mingels S, Dankaerts W, van Etten L, et al. Spinal postural variability relates to biopsychosocial variables in patients with cervicogenic headache. Sci Rep 2021;11(1):13783.
83. Mingels S, Dankaerts W, van Etten L, et al. Lower spinal postural variability during laptop-work in subjects with cervicogenic headache compared to healthy controls. Sci Rep 2021;11(1):5159.
84. Coronado RA, George SZ. The Central Sensitization Inventory and Pain Sensitivity Questionnaire: An exploration of construct validity and associations with widespread pain sensitivity among individuals with shoulder pain. Musculoskelet Sci Pract 2018;36:61-7.
85. Vaegter HB, Petersen KK, Mørch CD, et al. Assessment of CPM reliability: quantification of the within-subject reliability of 10 different protocols. Scand J Pain 2018;18(4):729-37.
86. Costa YM, de Araújo-Júnior ENS, Fiedler LS, et al. Reproducibility of quantitative sensory testing applied to musculoskeletal orofacial region: Site and sex differences. Eur J Pain 2019;23(1):81-90.
87. Langham MC, Floyd TF, Mohler ER, et al. Evaluation of cuff-induced ischemia in the lower extremity by magnetic resonance oximetry. J Am Coll Cardiol 2010;55(6):598-606.
88. Nahman-Averbuch H, Nir RR, Sprecher E, et al. Psychological Factors and Conditioned Pain Modulation: A Meta-Analysis. Clin J Pain 2016;32(6):541-54.
89. Graeff P, Itter A, Wach K, et al. Inter-Individual Differences Explain More Variance in Conditioned Pain Modulation Than Age, Sex and Conditioning Stimulus Intensity Combined. Brain Sci 2021;11(9)1186.

8. FIGURE LEGEND

For peer review only - http://bmjopen.bmj.com/site/about/guidelines.xhtml

Figure 1. Visualisation of the composition of the PMP (PMP = Pain Modulation Profile; CPM = Conditioned Pain Modulation; TSP = Temporal Summation of Pain; PPT = Pressure Pain Threshold).

Figure 2. Visualisation of the experimental protocol regarding TSP (PP = PinPrick; V1 = ophthalmic branch trigeminal nerve; min = minutes).

Figure 3. Visualisation of the experimental protocol regarding CPM (PPT = Pressure Pain Threshold (kPa/cm²); PPTo = Pressure Pain Tolerance (kPa/cm²); Sub = Suboccipital; L = Left; R = Right; CPA = Cuff Pressure Algometry non-dominant calf muscle (kPa); s = seconds; min = minutes).

Figure 4. Visualisation of the experimental protocol regarding widespread pain (PPT = Pressure Pain Threshold (kPa/cm²); Sub = Suboccipital; L = Left; R = Right; Tib = Tibialis anterior).

For peer review only

For peer review only - http://bmjopen.bmj.com/site/about/guidelines.xhtml

FIGURE 1

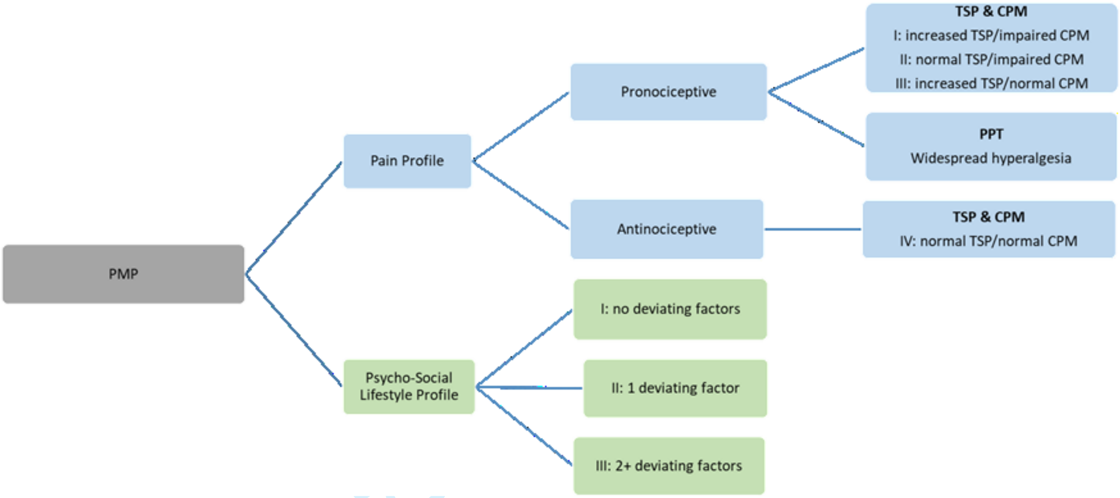

Figure 1. Visualisation of the composition of the PMP (PMP = Pain Modulation Profile; CPM = Conditioned Pain Modulation; TSP = Temporal Summation of Pain; PPT = Pressure Pain Threshold).

FIGURE 2

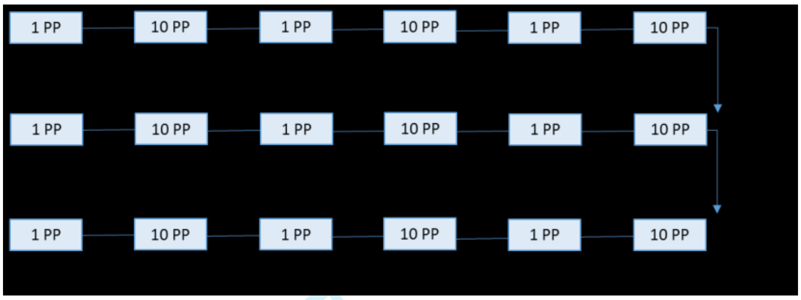

Figure 2. Visualisation of the experimental protocol regarding TSP (PP = PinPrick; V1 = ophthalmic branch trigeminal nerve; min = minutes).

For peer review only

FIGURE 3

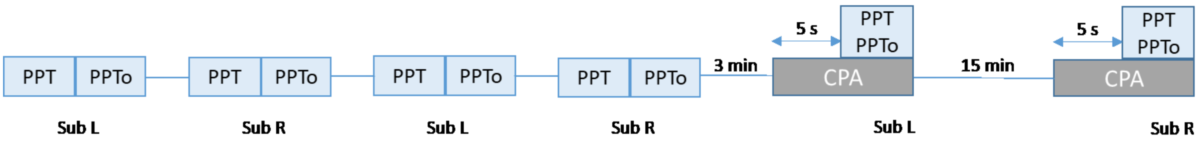

Figure 3. Visualisation of the experimental protocol regarding CPM (PPT = Pressure Pain Threshold (kPa/cm²); PPTo = Pressure Pain Tolerance (kPa/cm²); Sub = Suboccipital; L = Left; R = Right; CPA = Cuff Pressure Algometry non-dominant calf muscle (kPa); s = seconds; min = minutes).

For peer review only

FIGURE 4

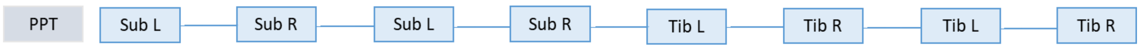

Figure 4. Visualisation of the experimental protocol regarding widespread pain (PPT = Pressure Pain Threshold (kPa/cm²); Sub = Suboccipital; L = Left; R = Right; Tib = Tibialis anterior).

For peer review only

SUPPLEMENTARY FILE

Supplement 1 – Checklist participant recruitment

The criteria that will be used to in- and exclude participants are summarized in Table S.1.

Table S.1 Summary of inclusion- and exclusion criteria for participants with CeH.

**Inclusion**	Caucasian males, females between 18-55 years
	Dutch-speaking
	Fulfilment of the diagnostic criteria for secondary CeH conform the ICHD-3 or CHISG (1,2)
	Diagnostic conformation by a neurologist
	Normal cognitive capacity (Mini Mental State Examination test score of 30).
	Willing to participate
**Exclusion**	Any other headache type or headache-related disorder
	Participation in another study related to headache
	Pregnancy
	Smoking
	First headache onset > 50 years
	Spinal surgery, head/neck trauma, headache-related physiotherapy ≤ 4 weeks prior the study
	Post-dural punction 2 weeks prior to the study
	Cognitive limitations (Mini Mental State Examination test score < 30)
	Confounding pathologies
	- Musculoskeletal: hernia, disk prolapse, congenital spinal deviations, TMD, hypermobility (Beighton ≥ 4)
	- Neurological: MS, Parkinson, epilepsy, CVA, myelopathy, myopathy, Benign Paroxysmal Position Vertigo, neurodegeneration, meningitis, encephalitis, dystonia, visual disorder
	- Endocrine - System: FM, CFS, rheumatoid arthritis, infectious/inflammatory diseases, sinusitis
	- Vascular: dysregulation blood pressure, postural hypotension, vertebra-basilar symptoms
	- Psychiatric
	- Medication-overuse: ergotamine, NSAID's, opioids, acetylsalicylic acid, triptans, simple analgesics (> 10 days/month for > 3 months)
	- Withdrawal of alcohol, caffeine or medication
	- Other: orthodontics, sleep apnoea

ICHD = International Classification Headache Disorders; CHISG = Cervicogenic Headache International Study Group Diagnostic; NSAID = Non Steroid Anti Inflammatory; MS = Multiple Sclerosis; CVA = Cerebro-Vascular Accident; FM = Fibromyalgia; CFS = Chronic Fatigue Syndrome; TMD = Temporo-mandibular disorder.

For peer review only

Supplement 2 – Time schedule

The time schedule for the feasibility study is visualized via Figure S.2.

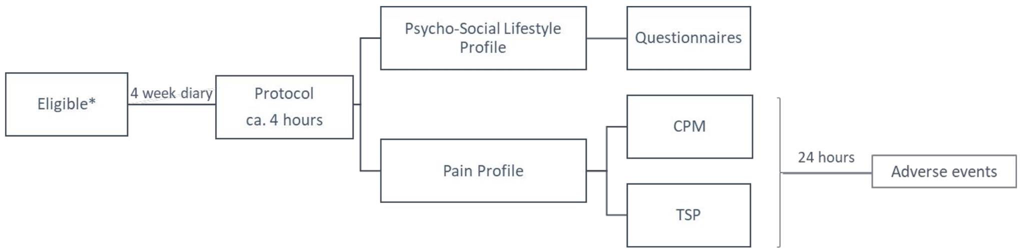

Figure S.2. Visualisation of the time schedule for the feasibility study (* = estimated time to recruit at least 12 people with CeH = 3 months based on previous experience; CPM = Conditioned Pain Modulation; TSP = Temporal Summation of Pain).

For peer review only - http://bmjopen.bmj.com/site/about/guidelines.xhtml

Supplement 3 – Conditioned pain modulation – widespread hyperalgesia

Figure S.3 provides a summary of the second CPM protocol.

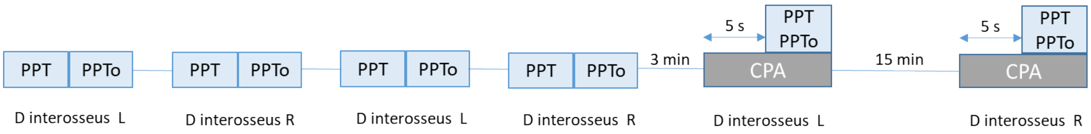

Figure S.3. Visualisation of the experimental protocol regarding CPM (PPT = Pressure Pain Threshold (kPa/cm²); PPTo = Pressure Pain Tolerance (kPa/cm²); D = Dorsal; L = Left; R = Right; CPA = Cuff Pressure Algometry non-dominant calf muscle (kPa); s = seconds; min = minutes).

Test stimulus: Mechanical pressure pain thresholds and tolerance

Measurements will be performed 15 minutes after the CPM with the testing stimulus applied at the suboccipital muscles. Pressure pain threshold (PPT) and pressure pain tolerance (PPTo) (kPa/cm²) will be measured at the left and right muscle belly of the first dorsal interosseous muscle using an electronic pressure algometer (Somedic AB, Stockholm, Sweden) (3,4). Pressure will be perpendicularly applied directly on the midpoint of the muscle belly, starting at 0 to maximal 1000 kPa, using a 1 cm² probe with a slope of 30 kPa/s. (5,6). Participants will be instructed to push the stop-button when the sensation of pressure first elicits pain (PPT), and when the maximum amount of pressure that could be tolerated is reached (PPTo).Intrarater reliability of the first dorsal interosseous muscle is excellent in healthy participants (ICC 0.91) (3).

Two repeated measurements at baseline, and two measurements parallel with the conditioning stimulus will be performed (Figure C.1). Averages of the extracted parameters will be used for further analysis (7).

Conditioning stimulus: Cuff pressure algometry

Cuff pressure tolerance (kPa) will be measured at the non-dominant lower leg. A cuff (Nocitech, Aalborg University, Denmark) will be placed around the bare non-dominant calf muscle, mounted with 8-cm distance between its upper border and the tibial tuberosity (8,9). Upper and lower borders of the cuff will be marked on the skin to ensure the cuff does not move between stimulations. Pressure tolerance will be determined through the maximal amount of pressure that can be tolerated. Intensity of the cuff pressure for the conditioning stimulus will be predefined as 70% of the pressure tolerance on the non-dominant leg (9,10). Computer-controlled cuff algometry (LabBench® CPAR+ instrument) shows an excellent intra-rater reliability (ICC 0.89) in healthy subjects (11,12). Test-retest reliability is good (ICCs 0.74 – 0.87) in healthy subjects (8).

For peer review only - http://bmjopen.bmj.com/site/about/guidelines.xhtml

Conditioned pain modulation (CPM)

CPM will be assessed 3 minutes after determining cuff pressure tolerance. The pressure (conditioning stimulus) will be kept constant throughout the CPM protocol (9,10). Five seconds after inflation of the cuff, PPT and PPTo will be reassessed as described above. Participants will be informed that the conditioning stimulus will be moderately painful, but that they should focus their attention on the test stimulus. The procedure described above will be sequentially and twice executed, i.e. once with the PPT-measurement at the left first dorsal interosseous muscle, and once with the PPT-measurement at the right first dorsal interosseous muscle as test stimulus. A 15-minute-interval will be provided between the CPM-measurements since CPM has a short lasting (< 15 minutes) hypoalgesic effect (Figure 2) (14,15).

At the moment, there is no consensus on a normal CPM effect. We propose a method described by Vaegter et al. (2016) to categorize the CPM response as normal or impaired based on the within-subject coefficient of variation (= within-subject SD/within-subject mean) in PPT (7). Participants will be profiled as having an impaired CPM if the CPM response is less than or equal to the normal within-subject coefficient of variation in PPT between 2 repeated assessments, and as having normal CPM if the CPM response is greater than the normal variation plus the upper limit of the 95% confidence interval (7).

For peer review only - http://bmjopen.bmj.com/site/about/guidelines.xhtml

Supplement 4 – Summary of the primary outcomes

Table S.4 provides a summary of the primary outcomes to estimate feasibility.

Table S.4. Summary of the adapted feasibility metrics and objectives (15,16).

Metric	Objectives	Outcome
**Process**	Determine recruitment and/or refusal rates per month	Percentage
Assess feasibility of processes key for future studies	Determine premature session endings and reasons(s) (e.g. pain, discomfort)	Percentage
	Operational feasibility (User Experience Questionnaire on usefulness, satisfaction, ease of learning, ease of use, and user experience after testing) (17)	Scores on a 7-point Likert scale converted to values ranging between < -3 (bad evaluation) and > 3 (extremely good evaluation)
**Resource**	Demonstrate appropriate eligibility criteria	Percentage and Qualitative
Assess time and resource problems that might occur in future studies	Estimate barriers/refusals to participation	Percentage and Qualitative
	Estimate access to/cost of equipment, space, time	Estimate cost/participant (i.e. staff, equipment, facility overheads)
	Clinician training needs and competence	Staff training requirements (time, frequency)
**Management**	Estimate equipment usage (e.g. availability)	Qualitative
Assess potential human and data management problems	Determine processing time for data collection	Time to mail data collection, time to complete outcome assessment
	Data collection and analysis – software appropriateness	Qualitative (identify requirements)
**Scientific**	Estimate challenges perceived/experienced by researcher	Qualitative
Assess safety, burden, data collection and response	Determine adverse events and safety procedure (e.g. local pain)	Percentage and Qualitative
	Burden of research	Percentage and Qualitative (feedback from participants before/during/after measurements)
	Determine appropriateness of target group	Qualitative (feedback from participants)
	Determine acceptability to participants	Qualitative (feedback from participants)
	Estimate outcome measures SD to determine future sample size	Statistical analysis of CPM (Pain Profile) to estimate future sample size

CPM = Conditioned Pain Modulation; SD = Standard Deviation.

For peer review only

For peer review only - http://bmjopen.bmj.com/site/about/guidelines.xhtml

References

1. Headache Classification Committee of the International Headache Society (IHS) The International Classification of Headache Disorders, 3rd edition. Cephalalgia 2018;38(1):1-211.
2. Fredriksen TA, Antonaci F, Sjaastad O. Cervicogenic headache: too important to be left un-diagnosed. J Headache Pain 2015;16:6.
3. Chesterton LS, Sim J, Wright CC, et al. Interrater reliability of algometry in measuring pressure pain thresholds in healthy humans, using multiple raters. Clin J Pain 2007;23(9):760-6.
4. Bogduk N. The neck and headaches. Neurol Clin 2014;32(2):471-87.
5. Alburquerque-Sendín F, Madeleine P, Fernández-de-Las-Peñas C, et al. Spotlight on topographical pressure pain sensitivity maps: a review. J Pain Res 2018;11:215-25.
6. Mingels S, Dankaerts W, van Etten L, et al. Exploring multidimensional characteristics in cervicogenic headache: Relations between pain processing, lifestyle, and psychosocial factors. Brain Behav 2021;11(10):e2339.
7. Vaegter HB, Graven-Nielsen T. Pain modulatory phenotypes differentiate subgroups with different clinical and experimental pain sensitivity. Pain 2016;157(7):1480-8.
8. Graven-Nielsen T, Vaegter HB, Finocchietti S, et al. Assessment of musculoskeletal pain sensitivity and temporal summation by cuff pressure algometry: a reliability study. Pain 2015;156(11):2193-202.
9. Cummins TM, Kucharczyk MM, Graven-Nielsen T, et al. Activation of the descending pain modulatory system using cuff pressure algometry: Back translation from man to rat. Eur J Pain 2020;24(7):1330-8.
10. Hoegh M, Petersen KK, Graven-Nielsen T. Effects of repeated conditioning pain modulation in healthy volunteers. Eur J Pain. 2018;22(10):1833-43.
11. Inventors'Way ApS. Cuff Pressure Algometry [online]. 2023. https://nocitech.com (accessed April 15th 2023).
12. Kvistgaard Olsen J, Fener DK, Wæhrens EE, et al. Reliability of Pain Measurements Using Computerized Cuff Algometry: A DoloCuff Reliability and Agreement Study. Pain Pract 2017;17(6):708-17.
13. Vaegter HB, Handberg G, Graven-Nielsen T. Similarities between exercise-induced hypoalgesia and conditioned pain modulation in humans. Pain 2014;155(1):158-67.
14. Vaegter HB, Handberg G, Jørgensen MN, et al. Aerobic exercise and cold pressor test induce hypoalgesia in active and inactive men and women. Pain Med 2015;16(5):923-33.
15. Learmonth YC, Motl RW. Important considerations for feasibility studies in physical activity research involving persons with multiple sclerosis: a scoping systematic review and case study. Pilot Feasibility Stud 2018;4:1.
16. Thorpe N, Harniess P, Main E, et al. Feasibility, safety and acceptability of select outcome measures in a physiotherapy study protocol for boys with haemophilia. Pilot Feasibility Stud 2021;7(1):105.
17. Laugwitz B. Construction and Evaluation of a User Experience Questionnaire. HCI and Usability for Education and Work. Heidelberg: Springer Berlin Heidelberg 2008

For peer review only

For peer review only - http://bmjopen.bmj.com/site/about/guidelines.xhtml

SPIRIT CHECKLIST

Table 1. SPIRIT 2013 Checklist: Recommended items to address in a clinical trial protocol and related documents (1)*

Section/item	Item	Description	Page
**Administrative information**			
Title	1	Descriptive title identifying the study design, population, interventions, and, if applicable, trial acronym	1
Trial registration	2a	Trial identifier and registry name. If not yet registered, name of intended registry	13
	2b	All items from the World Health Organization Trial Registration Data Set	13
Protocol version	3	Date and version identifier	1
Funding	4	Sources and types of financial, material, and other support	16
Roles and responsibilities	5a	Names, affiliations, and roles of protocol contributors	1,16
	5b	Name and contact information for the trial sponsor	16
	5c	Role of study sponsor and funders, if any, in study design; collection, management, analysis, and interpretation of data; writing of the report; and the decision to submit the report for publication, including whether they will have ultimate authority over any of these activities	16
	5d	Composition, roles, and responsibilities of the coordinating centre, steering committee, endpoint adjudication committee, data management team, and other individuals or groups overseeing the trial, if applicable (see Item 21a for data monitoring committee)	n/a
**Introduction**			
Background and rationale	6a	Description of research question and justification for undertaking the trial, including summary of relevant studies (published and unpublished) examining benefits and harms for each intervention	3,4
	6b	Explanation for choice of comparators	n/a
Objectives	7*	Specific objectives or research questions for pilot trial	4
Trial design	8	Description of trial design including type of trial (eg, parallel group, crossover, factorial, single group), allocation ratio, and framework (eg, superiority, equivalence, noninferiority, exploratory)	4,5
**Methods: Participants, interventions, and outcomes**			
Study setting	9	Description of study settings (eg, community clinic, academic hospital) and list of countries where data will be collected. Reference to where list of study sites can be obtained	5,6
Eligibility criteria	10	Inclusion and exclusion criteria for participants. If applicable, eligibility criteria for study centres and individuals who will perform the interventions (eg, surgeons, psychotherapists)	5, Appendix A
Interventions	11a	Interventions for each group with sufficient detail to allow replication, including how and when they will be administered	6,7 / 9-12 / Appendix B Figures 1-4
	11b	Criteria for discontinuing or modifying allocated interventions for a given trial participant (eg, drug dose change in response to harms, participant request, or improving/worsening disease)	6,8

For peer review only - http://bmjopen.bmj.com/site/about/guidelines.xhtml

	11c	Strategies to improve adherence to intervention protocols, and any procedures for monitoring adherence (eg, drug tablet return, laboratory tests)	n/a
	11d	Relevant concomitant care and interventions that are permitted or prohibited during the trial	6
Outcomes	12*	Completely defined prespecified assessments or measurements to address each pilot trial objective, including how and when they were assessed	6-7 / 9-12 / Appendix B
Participant timeline	13	Time schedule of enrolment, interventions (including any run-ins and washouts), assessments, and visits for participants. A schematic diagram is highly recommended (see Figure)	6, Appendix C
Sample size	14*	Rationale for numbers in the pilot trial	8,13
Recruitment	15	Strategies for achieving adequate participant enrolment to reach target sample size	14,15
**Methods: Assignment of interventions (for controlled trials)**			
Allocation:			
Sequence generation	16a	Method of generating the allocation sequence (eg, computer-generated random numbers), and list of any factors for stratification. To reduce predictability of a random sequence, details of any planned restriction (eg, blocking) should be provided in a separate document that is unavailable to those who enrol participants or assign interventions	n/a
Allocation concealment mechanism	16b	Mechanism of implementing the allocation sequence (eg, central telephone; sequentially numbered, opaque, sealed envelopes), describing any steps to conceal the sequence until interventions are assigned	n/a
Implementation	16c	Who will generate the allocation sequence, who will enrol participants, and who will assign participants to interventions	n/a
Blinding (masking)	17a	Who will be blinded after assignment to interventions (eg, trial participants, care providers, outcome assessors, data analysts), and how	n/a
	17b	If blinded, circumstances under which unblinding is permissible, and procedure for revealing a participant's allocated intervention during the trial	n/a
**Methods: Data collection, management, and analysis**			
Data collection methods	18a	Plans for assessment and collection of outcome, baseline, and other trial data, including any related processes to promote data quality (eg, duplicate measurements, training of assessors) and a description of study instruments (eg, questionnaires, laboratory tests) along with their reliability and validity, if known. Reference to where data collection forms can be found, if not in the protocol	6-12
	18b	Plans to promote participant retention and complete follow-up, including list of any outcome data to be collected for participants who discontinue or deviate from intervention protocols	5,6,8
Data management	19	Plans for data entry, coding, security, and storage, including any related processes to promote data quality (eg, double data entry; range checks for data values). Reference to where details of data management procedures can be found, if not in the protocol	12,13
Statistical methods	20a*	Methods used to address each pilot trial objective whether qualitative or quantitative	13

For peer review only - http://bmjopen.bmj.com/site/about/guidelines.xhtml

	20b	Methods for any additional analyses (eg, subgroup and adjusted analyses)	n/a
	20c	Definition of analysis population relating to protocol non-adherence (eg, as randomised analysis), and any statistical methods to handle missing data (eg, multiple imputation)	n/a
**Methods: Monitoring**			
Data monitoring	21a	Composition of data monitoring committee (DMC); summary of its role and reporting structure; statement of whether it is independent from the sponsor and competing interests; and reference to where further details about its charter can be found, if not in the protocol. Alternatively, an explanation of why a DMC is not needed	15
	21b	Description of any interim analyses and stopping guidelines, including who will have access to these interim results and make the final decision to terminate the trial	n/a
Harms	22	Plans for collecting, assessing, reporting, and managing solicited and spontaneously reported adverse events and other unintended effects of trial interventions or trial conduct	5,6,8
Auditing	23	Frequency and procedures for auditing trial conduct, if any, and whether the process will be independent from investigators and the sponsor	n/a
**Ethics and dissemination**			
Research ethics approval	24	Plans for seeking research ethics committee/institutional review board (REC/IRB) approval	5
Protocol amendments	25	Plans for communicating important protocol modifications (eg, changes to eligibility criteria, outcomes, analyses) to relevant parties (eg, investigators, REC/IRBs, trial participants, trial registries, journals, regulators)	6
Consent or assent	26a	Who will obtain informed consent or assent from potential trial participants or authorised surrogates, and how (see Item 32)	5
	26b	Additional consent provisions for collection and use of participant data and biological specimens in ancillary studies, if applicable	n/a
Confidentiality	27	How personal information about potential and enrolled participants will be collected, shared, and maintained in order to protect confidentiality before, during, and after the trial	12,13
Declaration of interests	28	Financial and other competing interests for principal investigators for the overall trial and each study site	16
Access to data	29	Statement of who will have access to the final trial dataset, and disclosure of contractual agreements that limit such access for investigators	12,13
Ancillary and post-trial care	30	Provisions, if any, for ancillary and post-trial care, and for compensation to those who suffer harm from trial participation	n/a
Dissemination policy	31a	Plans for investigators and sponsor to communicate trial results to participants, healthcare professionals, the public, and other relevant groups (eg, via publication, reporting in results databases, or other data sharing arrangements), including any publication restrictions	n/a
	31b	Authorship eligibility guidelines and any intended use of professional writers	n/a
	31c	Plans, if any, for granting public access to the full protocol, participant-level dataset, and statistical code	n/a

For peer review only - http://bmjopen.bmj.com/site/about/guidelines.xhtml

**Appendices**			
Informed consent materials	32	Model consent form and other related documentation given to participants and authorised surrogates	Appendix D
Biological specimens	33	Plans for collection, laboratory evaluation, and storage of biological specimens for genetic or molecular analysis in the current trial and for future use in ancillary studies, if applicable	n/a

n/a = Not Applicable; * = item numbers 12, 14, and 20a were replaced by items 6a, 7a, and 12a from the CONSORT-extensions (2) checklist based on the article of Thabane and Lancaster (2019) (3)

REFERENCES

1. Chan A-W, Tetzlaff JM, Altman DG, et al. SPIRIT 2013 Statement: Defining standard protocol items for clinical trials. Ann Intern Med 2013;158(3):200-7.
2. Eldridge SM, Chan CL, Campbell MJ, et al. CONSORT 2010 statement: extension to randomised pilot and feasibility trials. BMJ 2016;355:i5239.
3. Thabane, L., Lancaster, G. A guide to the reporting of protocols of pilot and feasibility trials. Pilot Feasibility Stud 2019;5:37.

For peer review only

BMJ Open

Cross-Sectional Experimental Assessment of Pain Modulation as part of Multidimensional Profiling of People with Cervicogenic Headache: Protocol for a Feasibility Study

Journal:	*BMJ Open*
Manuscript ID	bmjopen-2023-074743.R1
Article Type:	Protocol
Date Submitted by the Author:	15-Nov-2023
Complete List of Authors:	Mingels, Sarah; Katholieke Universiteit Leuven, Department of Rehabilitation Sciences; Hasselt University<br>Granitzer, Marita; Hasselt University<br>Schmid, Annina ; University of Oxford, Nuffield Department of Clinical Neurosciences; The University of Queensland, School of Health and Rehabilitation Sciences<br>Graven-Nielsen, Thomas ; Aalborg University<br>Dankaerts, Wim ; KU Leuven
**Primary Subject Heading**:	Research methods
Secondary Subject Heading:	Diagnostics
Keywords:	Feasibility Studies, Chronic Pain, Neurological pain < NEUROLOGY, Risk Factors

SCHOLARONE™
Manuscripts

Cross-Sectional Experimental Assessment of Pain Modulation as part of Multidimensional Profiling of People with Cervicogenic Headache: Protocol for a Feasibility Study

Sarah Mingels[1,2*], Marita Granitzer[2], Annina Schmid[3], Thomas Graven-Nielsen[4], and Wim Dankaerts[1]

[1] Musculoskeletal Research Unit, Department of Rehabilitation Sciences, Faculty of Kinesiology and Rehabilitation Sciences, Leuven University, Leuven, Belgium

[2] REVAL Rehabilitation Research Centre, Biomedical Research Institute, Faculty of Rehabilitation Sciences, Hasselt University, Hasselt, Belgium

[3] Nuffield Department of Clinical Neurosciences, University of Oxford, Oxford, United Kingdom

[4] Center for Neuroplasticity and Pain (CNAP), Department of Health Science and Technology, Aalborg University, Aalborg, Denmark

* corresponding author: sarah.mingels@kuleuven.be

For peer review only

Protocol version: 3

Protocol date: June 3rd 2024

For peer review only - http://bmjopen.bmj.com/site/about/guidelines.xhtml

ABSTRACT

Background. An endogenous pain modulation profile, reflecting anti-nociceptive and pro-nociceptive mechanisms, may help to direct management by targeting the involved pain-mechanism. For individuals with cervicogenic headache (CeH) the characteristics of such profiles were never investigated. However, the individual nature of experiencing pain demands profiling within a multidimensional framework including psycho-social-lifestyle characteristics. The objective of the current protocol is to assess the pain modulation profile, which includes psycho-social-lifestyle characteristics among people with CeH.

Methods and analysis. A protocol is described to map pain modulation profiles in people with CeH. A cross-sectional non-randomized experimental design will be used to assess feasibility of mapping these profiles. The pain modulation profile is composed based on results on the Depression, Anxiety, Stress Scale (DASS-21), Pittsburgh Sleep Quality Index (PSQI), Headache Impact Test (HIT-6), and on responses to temporal summation of pain (pinprick), conditioned pain modulation, and widespread hyperalgesia (mechanical pressure pain threshold and cuff algometry). Primary analyses will report results relating to outcomes on feasibility. Secondary analyses will involve an analysis of proportions (%) of the different Psycho-Social Lifestyle Profiles and Pain Profiles.

Ethics and dissemination. Ethical approval was granted by the Ethics Committee Research UZ/KU Leuven (Registration number B3222024001434) on May 30th 2024. Results will be published in peer-reviewed journals, at scientific conferences and, through press releases.

Strengths and limitations of this study

- An innovative protocol is developed to assess facilitated pain processing in people with cervicogenic headache
- Multiple dimensions (pain processing, psycho-social-lifestyle) of cervicogenic headache will be explored
- No biomarkers exist to determine facilitated pain processing. The pain status can only be estimated through proxy measures such as the conditioned pain modulation and widespread hyperalgesia
- If successful, the protocol can be adapted to increase clinical applicability

For peer review only - http://bmjopen.bmj.com/site/about/guidelines.xhtml

1. Introduction

Cervicogenic headache (CeH) is a secondary headache attributed to dysfunctions of the cervical spine (1). Underdiagnosed and undertreated, CeH might evolve into a chronic state, increasing the odds of absenteeism and disability (2,3). It is generally accepted to adopt a multidimensional approach in the management of pain (4,5) Interestingly though, such an approach is not recommended when managing people with CeH (6). Results from a Delphi-study indicated that lifestyle advice, pain education, and cognitive therapy were considered not to be relevant in the management of CeH (6). Currently, the non-pharmacological management of CeH mainly focusses on targeting the musculoskeletal dysfunctions of the (upper) cervical spine (6-10). However, a meta-analysis with pooled outcome parameters showed inconsistent results of such management on headache intensity, frequency, and related disability (11). Although non-pharmacological interventions may play an important role in managing CeH, there is currently limited scientific evidence to fully support these interventions (11). It has been stated previously that some therapeutic interventions are not appropriate for all people with CeH (12).

When CeH is merely mediated by a peripheral nociceptive source (i.e. musculoskeletal dysfunction of the upper-cervical spine), also known as bottom-up source, this could be managed by addressing the dysfunction through manual therapy and/or specified exercises (13). Such management is likely to be inefficient if signs of facilitated central pain mechanisms are present. In such state, exclusively aiming at the peripheral source might act as a nociceptive stimulus maintaining facilitated central pain processing (13,14) The pathophysiology of CeH can thus generally be explained by (a) CeH caused by an exclusive peripheral input, or (b) CeH caused by peripheral input and maintained by sensitization processes. It has been argued that characterizing the involved dominant central pain mechanism might provide valuable information to increase therapy efficacy (15). Central pain mechanisms such as pro- or antinociceptive mechanisms (16), have however not extensively been examined in people with CeH. Results from a study on pain processing, showing lower extra-cephalic and cephalic pressure pain thresholds in people with CeH compared to healthy controls, might indicate a dysfunctional central pain mechanism (17,18).

Dynamic protocols, to evoke inhibition or facilitation of pain, have been designed to evaluate endogenous pain modulation, and to define a pro- or antinociceptive central pain mechanism. Protocols to assess pain inhibition evaluate the conditioned pain modulation (CPM) paradigm, and pain facilitation assess temporal summation of pain (TSP) (16). The term CPM was created for psychophysical protocols that explore diffuse noxious inhibitory control in humans (19). The latter manifests as the inhibition of wide-dynamic-range neuronal activity by descending adrenergic pathways (19). CPM is measured by comparing pain induced by a test stimulus, with pain induced by the same test stimulus either during (parallel) or after (sequential) a conditioning stimulus. TSP is a

For peer review only - http://bmjopen.bmj.com/site/about/guidelines.xhtml

nociceptive mediated process which is considered to reflect advanced spinal synaptic facilitation in the dorsal horn (i.e. behavioural correlate of wind-up) (20,21). TSP is a measured by comparing pain ratings between a single noxious stimulus and repeated equal-intensity noxious stimuli at a specific frequency. A third proxy to examine facilitated central pain mechanisms is widespread hyperalgesia (22).

Assessing endogenous pain modulation and phenotyping (23-25) in people with CeH within a multidimensional framework is needed in the context of therapy-unresponsiveness. Despite the well-known pathophysiology of CeH, the number of non-responders to non-pharmacological non-invasive therapy amounts to 25%, and self-reported effectiveness of manual therapy is rated as 36% (26,27). Such therapy-unresponsiveness has in other musculoskeletal disorders been related to inadequate health literacy, neural sensitivity, or augmented pain processing in the central nervous system (26, 27). Therefore, a Pain Modulation Profile (PMP) needs to be composed. The PMP includes, besides measurements to analyse central pain mechanisms, also measurements of potential influential factors (e.g. demographic, psycho-social, lifestyle) that can influence such mechanisms. These factors might explain some of the inter-individual variability in pain perception, and therefore, possibly also play a role in CPM (28). Nociceptive inputs activate complex interactions among cortical regions that are also active in cognitive, emotional, and reward functions. These regions influence serotonergic and noradrenergic descending pain modulatory systems bimodally via processes between the periaqueductal grey, rostral ventromedial medulla and pontine noradrenergic nuclei, ultimately facilitating or inhibiting nociceptive input. Descending pain modulatory pathways can therefore be stimulated from the top down, i.e. from the brain to brainstem, by psycho-social interventions (29-31). Further, also lifestyle factors can influence pain processing. Preliminary evidence is provided that an active lifestyle could reduce spinal nociception (i.e. nociceptive flexion reflex) in healthy adults (30). Animal studies indicated that regular physical activity influences central cellular processes (i.e. decrease neuronal excitability, alter neuro-immune signalling, increase release of endogenous opioids and serotonin in the descending pain modulatory pathways) involved in dysregulation of endogenous pain modulatory system, and development of chronic pain (32-34). Additionally, one night of total sleep deprivation impaired descending pain modulatory pathways, facilitated spinal neuronal excitability, and facilitated peripheral pain mechanisms in heathy participants (35).

The objective of the current paper is to describe a study protocol which aims to feasibly assess the PMP as part of multidimensional profiling among people with CeH. The advantage of determining such PMP is that it offers the possibility to address the involved pain-mechanism(s) within a multidimensional framework.

For peer review only - http://bmjopen.bmj.com/site/about/guidelines.xhtml

2. Methods and analysis

2.1 Design

A first step toward achieving the objective was to describe a protocol to feasibly assess PMPs among participants with CeH. The next step will be to conduct a feasibility study. The protocol (i.e. eligibility criteria, set-up, analyses of outcomes) will be adapted based on the findings and feedback gathered from the feasibility study. Relevant adaptations will be communicated with the principal investigator (prof. W Dankaerts) and the Ethics Committee Research UZ / KU Leuven.

The measurements and instruments were selected based on a literature review, and after consensus with an expert panel (n = 5) consisting of experts in neurophysiology (MG), neuroscience (AS), biomedical Science, engineering and medical science (TGN), and musculoskeletal rehabilitation (WD, SM) (October 2022 – February 2023).

A description of the measurements, outcomes, instruments, and procedure is outlined below (Table 1 and paragraph 2.5). Enrolled participants will complete the protocol, and will be profiled based on their PMP. This profile comprises a Pain Profile and Psycho-Social Lifestyle Profile. A cross-sectional non-randomized design will be used to determine if PMPs can be feasibility assessed.

For peer review only

Table 1. Summary of the measurements, outcomes, and instruments to feasibly assess PMPs.

**Primary outcome**	**Instrument**	**Measurement**
Feasibility	Personalized questionnaire	Process metrics<br>Resource metrics<br>Management metrics<br>Scientific metrics
	UEQ	Operational feasibility
**Profiling related outcome**	**Instrument**	**Measurement**
Sociodemographics	Questionnaire	Age – Gender – Body mass index<br>Socio-economic status
Headache characteristics	Headache-diary	Intensity – Frequency – Duration – Medication intake
Pain Profile	Pressure cuff	Conditioned Pain Modulation (conditioning stimulus)
	Algometer	Conditioned Pain Modulation (test stimulus)<br>Widespread hyperalgesia
	Pinprick stimulator	Temporal Summation
Psycho-Social Lifestyle Profile	DASS-21	Depression, Anxiety, Stress
	PSQI	Sleep Quality
	HIT-6	Quality of Life

UEQ = User Experience Questionnaire (36); DASS-21 = Depression, Anxiety, Stress Scale; PSQI = Pittsburgh Sleep Quality Index; HIT-6 = Headache Impact Test.

2.2 Study population and setting

Participants with CeH will be screened and recruited via the neurological staff of the headache department of the AZ Vesalius hospitals (Tongeren and Bilzen, Belgium).

A detailed summary of the study population's inclusion and exclusion criteria is provided in table S1. A four-week headache-diary will be distributed among the enrolled participants to collect information

For peer review only - http://bmjopen.bmj.com/site/about/guidelines.xhtml

on the following headache characteristics: intensity, frequency, duration, and medication intake. Eligible participants will need to read and sign the informed consent (collected by the principal researcher) before officially being enrolled. The experimental set-up will be at the Leuven University (Leuven, Belgium) and private practice of the principal researcher (Riemst, Belgium).

2.3 Patient and public Involvement

People with CeH will be involved in the feasibility study. This study aims to gather information on arguments to participate or decline to participate, reason(s) for premature ending, appropriateness of the measurements, and barriers, adverse events, or burden experienced by people with CeH. The information will be used to adapt the protocol. Feedback on possible adaptations will be provided.

2.4 Experimental protocol

A PMP, consisting of a Psycho-Social Lifestyle Profile and Pain Profile, will be composed (Figure 1). Three questionnaire will be used to estimate the Psycho-Social Lifestyle Profile. The Pain Profile will be interpreted based on three parameters (TSP, CPM, widespread hyperalgesia).

[Figure 1]

Assessment will be in a headache-free phase (= score of 0 on the Numeric Pain Rating Scale). Participants will be informed on possible adverse events, and asked to abstain from vigorous physical activity, taking analgesics, and caffeine-containing beverages 24 hours prior to the measurements. Prophylactic treatment(s) remain unchanged (1). Adverse events will be questioned after finalizing the test-procedures, and again after 24 hours. Measurements will be performed by the principal researcher (manual therapist, > 10 years clinical experience, PhD Physiotherapy & Rehabilitation Sciences) in a chronological order (Figures 2-4). The entire procedure is estimated to amount to 4 hours. An overview of the time schedule for the feasibility study is provide in figure S1.

2.4.1 Psycho-Social Lifestyle Profile - Questionnaires

The Pittsburgh Sleep Quality Index (PSQI), Depression, Anxiety, Stress Scale (DASS-21), and Headache Impact Test (HIT-6) will be completed in a quiet room prior to the CPM, TSP, and Pressure Pain Threshold measurements. Thereafter the User Experience Questionnaire will be filled in. The principal researcher will be available in case of ambiguity about the questions/statements.

2.4.2 Pain Profile - Temporal Summation of Pain

Figure 2 provides a summary of the TSP protocol.

For peer review only - http://bmjopen.bmj.com/site/about/guidelines.xhtml

[Figure 2]

2.4.3 Pain Profile - Conditioned Pain Modulation

Figure 3 provides a summary of the CPM protocol which will be executed 15 minutes after the TSP measurements.

[Figure 3]

2.4.4 Pain Profile - Widespread hyperalgesia

Figure 4 provides a summary of the protocol to evaluate widespread hyperalgesia which will be executed 15 minutes after the CPM measurements (figure S2).

[Figure 4]

2.5 Measurements, outcomes, instruments, and procedure

2.5.1 Primary outcome – Feasibility

Feasibility of the protocol will be assessed using adapted feasibility metrics. These metrics include: process, resource, management, and scientific metrics (37,38). Details on the primary outcomes are summarized in table S2.

For peer review only

For peer review only - http://bmjopen.bmj.com/site/about/guidelines.xhtml

2.5.2 Profiling-related outcome – Psycho-Social Lifestyle Profile

Participants will be categorised in Psycho-Social Lifestyle Profile based on the number of deviating psychosocial-lifestyle factors deducted from scores on the PSQI, DASS-21, and HIT-6. Individual scores will be compared to normative data (17). Scores indicating at least: moderate depression, anxiety, stress (DASS-21), headache has a significant impact on daily life (HIT-6), and/or poor sleep quality (PSQI) will be considered as deviating.

2.5.2.1 Psycho-Social Lifestyle factors

Sleep quality will be assessed via the Dutch PSQI, a standardized, valid, and reliable self-reported one-month recall questionnaire (39,40). The index differentiates poor from good sleepers by measuring: subjective sleep quality, sleep latency, sleep duration, habitual sleep efficiency, sleep disturbances, use of sleeping medication, and daytime dysfunction. Scores on each of these components vary from 0 (‘No problem’) to 3 (‘Serious problem’). A maximum score exceeding 5/21 indicates poor sleep quality (41,42). See Mollayeva et al. (2016) for information on the psychometric properties (39).

The degree of *depression, anxiety, and stress* will be estimated by the Dutch DASS-21 (43,44). The DASS-21 is a self-reported, one-week recall questionnaire. Each of the three sub-scales contain seven items. The depression subscales assess dysphoria, hopelessness, devaluation of life, self-deprecation, lack of interest, anhedonia, and inertia. The anxiety subscale estimates autonomic arousal, skeletal muscle effects, situational anxiety, and subjective experience of anxious affect. The stress subscale evaluates difficulty in relaxing, nervous arousal, being easily upset, and impatience. Items are scored on a Likert-scale (0 = ‘Did not apply to me at all’, and 3 = ‘Applied to me very much or most of the time’). Scores of 14, 10, and 19 indicate at least moderate depression, anxiety, and stress, respectively. See Lovibond et al. (1995) for information on the psychometric properties (45).

Impact *of headache on quality of life* will be estimated by the Dutch HIT-6 (46,47). The HIT-6 evaluates the impact of headache on daily activities: the ability to function at work, school, home, and in social situations. Items are scored 6, 8, 10, 11, and 13 (6 = ‘Never’, 8 = ‘Rarely’, 10 = ‘Sometimes’, 11 = ‘Very often’, and 13 = ‘Always’). Scores exceeding 56 indicate headache has a significant impact on daily life (47,48). See Martin et al. (2004) and Kosinski et al. (2003) for information on the psychometric properties (47,49).

For peer review only - http://bmjopen.bmj.com/site/about/guidelines.xhtml

2.5.3 Profiling-related outcome – Pain Profile

Participants will be categorised in four Pain Profiles based on responses to the TSP and CPM (Figures 1-3):

P-P I: Pronociceptive (increased TSP/impaired CPM)

P-P II: Pronociceptive (normal TSP/impaired CPM)

P-P III: Pronociceptive (increased TSP/normal CPM)

P-P IV: Antinociceptive (normal TSP/normal CPM)

Each participant with CeH will be categorised into a Pain Profile per measurement location (TSP at the hand and face, CPM at the left and right suboccipital muscles) (50,51). Additional presence of widespread pain will be evaluated (Figure 4). The paragraphs below contain information on the definitions of increased/decreased TSP, impaired/normal CPM, and presence of widespread hyperalgesia.

2.5.3.1 Pinprick stimulus: Temporal summation of Pain (TSP)

The pinprick evoked Wind-Up Ratio (WUR) (Pinprick stimulator, MRC Systems GmbH - Medizintechnische Systeme) is suggested to evaluate TSP. Participants will be positioned in sitting. The WUR will be assessed at the bilateral (i.e. painful and pain-free sides) ophthalmic region (V1), and extra-segmentally on the thenar eminence of the dominant hand (50,52-54). WUR represents the quotient of pain intensity evoked by one single pinprick stimulus divided by the average pain evoked by 10 repetitive pinprick stimuli (52-54). We additionally propose to apply the SumSquare method as described by Allison et al. (2021) to quantifying TSP (55). The WUR is reported to lack sensitivity to detect differences in the magnitude of sensitisation. Most studies report little variability (ratio 1.6-1.7) in WUR measurements both in normal and hyperalgesic states (55).

For the measurements at the face and hand constant pinpricks of 128 mN and 256mN, respectively will be used (53). First, a single pinprick stimulus will be applied, followed by a series of 10 pinprick stimuli with a frequency of 1 Hz within an area of 1 cm^2 (51,53). The interval between the single, and repetitive stimuli will be 1 minute, or until all aftersensations have resolved. Participants will be asked to rate the pain of a single pinprick stimulus, and the average pain at the end of the series of 10 pinpricks using the 0-10 Numeric Pain Rating Scale (0 = no pain, 10 = the worst pain imaginable). The test order will be standardized as: dominant hand – left side face – right side face. This procedure will be repeated three times at each site, and three measurements per site will be averaged (Figure 2). We refer to the normative values provided by the German Research Network on Neuropathic Pain to determine normal or increased TSP at the face and hand (52). Test-retest reliability for pinprick induced TSP is moderate-to-good in healthy participants (ICC 0.51 – 0.61) (56).

For peer review only - http://bmjopen.bmj.com/site/about/guidelines.xhtml

2.5.3.2 Test stimulus: Mechanical pressure pain thresholds and tolerance

Measurements will be performed 15 minutes after the TSP measurements. Pressure pain threshold (PPT) and pressure pain tolerance (PPTo) (kPa/cm²) will be measured at the left and right suboccipital muscles using an electronic pressure algometer (Somedic AB, Stockholm, Sweden) (57). Pressure will be perpendicularly applied directly on the suboccipital muscles, starting at 0 to maximal 1000 kPa, using a 1 cm² probe with a slope of 30 kPa/s (17,58). Participants will be instructed to push the stop-button when the sensation of pressure first elicits pain (PPT), and when the maximum amount of pressure that could be tolerated is reached (PPTo). A trial will be performed once on the left shin (tibialis anterior muscle belly, approximately 2.5 cm lateral and 5 cm inferior to the tibial tubercle) before measuring the suboccipital muscles (59). Intrarater reliability of tibialis anterior PPT-measurements are good-to-excellent in people with CeH (ICC 0.82-0.92) (60).

Two repeated measurements at baseline, and two measurements parallel with the conditioning stimulus will be performed with (Figure 3). Averages of the extracted parameters will be used for further analysis (61). Test-retest reliability of PPT-measurements at the suboccipital muscles is good to excellent (ICC 0.84 – 0.93) (62). Intra-rater reliability of such PPT-measurements are moderate-to-good in people with CeH (ICC 0.69 - 0.87) (17,63).

2.5.3.3 Conditioning stimulus: Cuff pressure algometry

Cuff pressure tolerance (kPa) will be measured at the non-dominant lower leg. A cuff (Nocitech, Aalborg University, Denmark) will be placed around the bare non-dominant calf muscle, mounted with 8-cm distance between its upper border and the tibial tuberosity (64,65). Upper and lower borders of the cuff will be marked on the skin to ensure the cuff does not move between stimulations. Pressure tolerance will be determined through the maximal amount of pressure that can be tolerated. Intensity of the cuff pressure for the conditioning stimulus will be predefined as 70% of the pressure tolerance on the non-dominant leg (64-66). Computer-controlled cuff algometry (LabBench® CPAR+ instrument) shows an excellent intra-rater reliability (ICC 0.89) in healthy subjects (67,68). Test-retest reliability is good (ICCs 0.74 – 0.87) in healthy subjects (64).

2.5.3.4 Conditioned pain modulation (CPM)

CPM will be assessed 3 minutes after determining cuff pressure tolerance. The pressure (conditioning stimulus) will be kept constant throughout the CPM protocol (65,66). Five seconds after inflation of the cuff, PPT and PPTo will be reassessed as described above. Participants will be informed that the conditioning stimulus will be moderately painful, but that they should focus their attention on the test stimulus. The procedure described above will be executed twice sequentially, i.e. once with the PPT-measurement at the left suboccipital muscles, and once with the PPT-measurement at the right

For peer review only - http://bmjopen.bmj.com/site/about/guidelines.xhtml

suboccipital muscles as test stimulus. A 15-minute-interval will be provided between the CPM-measurements since CPM has a short lasting (< 15 minutes) hypoalgesic effect (Figure 3) (69-72).

At the moment, there is no consensus on a normal CPM effect. We propose a method described by Vaegter et al. (2016) to categorize the CPM response as normal or impaired based on the within-subject coefficient of variation (= within-subject SD/within-subject mean) in PPT. Participants will be profiled as having an impaired CPM if the CPM response is less than or equal to the normal within-subject coefficient of variation in PPT between 2 repeated assessments, and as having normal CPM if the CPM response is greater than the normal variation plus the upper limit of the 95% confidence interval (61).

2.5.3.5 Widespread hyperalgesia

Widespread hyperalgesia indicates facilitated central pain mechanisms (73). PPT and CPM measurements will be used to estimate widespread hyperalgesia.

PPTs at both symptomatic (bilateral suboccipital muscles) and distant pain-free (bilateral tibialis anterior muscles) areas will be used to determine widespread hyperalgesia. PPTs lower than the 95% confidence interval lower border bound of the normative PPTs will be considered decreased (= deviating) (17). PPTs at the suboccipitals will not be repeated since data are already collected within the context of CPM (cfr. paragraph 2.5.3.4). PPTs of the bilateral tibialis anterior muscle will be determined as described above (cfr. paragraph 2.5.3.2). The test order will be standardized as: left tibialis anterior – right tibialis anterior muscle. This procedure will be repeated two times, and measurements per site will be averaged. Averages of the extracted parameters will be used for further analysis (61). An extra-segmental *CPM measurement* will be performed as second proxy to estimate widespread hyperalgesia (figure S2).

For peer review only

2.6 Data Management plan

Data concerning feasibility, CPM, TSP, and widespread hyperalgesia will be electronically collected (.xlsx). Data gathered from the paper questionnaires (PSQI, DASS-21, HIT-6) will be transferred to excel files (.xlsx). Data will be protected by pseudonymising them, restricting access, and access only via the multifactor-authentication (KU Leuven Authenticator app). The principal researcher will complete the pseudonymising process and GDPR-questionnaire according to the KU Leuven policy. Storing is foreseen in a folder structure (OneDrive, Microsoft 365 Apps for enterprise) to secure: availability of data, confidentiality, and sharing data (https://icts.kuleuven.be/storagewijzer/nl). Finished data (i.e. the dataset) will be stored at the KU Leuven Research Data Repository (https://www.kuleuven.be/rdm/en/rdr/rdr). This platform enables archiving (at the end of the

For peer review only - http://bmjopen.bmj.com/site/about/guidelines.xhtml

project), uploading, describing, and sharing research data in a legal and controlled manner. Exclusively the principal researcher and principal investigator will have access to all data, other team members will only obtain restricted access. The latter implies that data are anonymous for these team members. All data will be kept for 10 years, conform the KU Leuven policy. Supportive arguments to keep personal data for 10 years include: verification of results and future research. Final versions of datasets will be preserved for the long-term on internal KU Leuven data storage facilities.

2.7 Statistics

Primary analyses will report results relating to outcomes on feasibility. Descriptive statistics and content analysis will be used to assess feasibility of the protocol. The feasibility study will provide useful information with regard to planning, testing study procedures (e.g. estimation of the recruitment rate, plausibility of multicentre collaborations, etc.), and investigating outcomes to support adequate sample size calculation (74). Data from the CMP and TSP measurements will be used to estimate post hoc sample size (G*Power 3.1.9.4, Kiel Germany) for a larger trial.

Individual PMPs of at least 12 participants with CeH will be mapped based on the methods outlined in sections 2.5.2 and 2.5.3. Secondary analyses will involve an analysis of proportions (%) of the different Psycho-Social Lifestyle Profiles and Pain Profiles. Currently no data are available in people with CeH, therefore a pragmatic approach is proposed based on the work of Julious (2005) who recommended 12 participants as the minimum for pilot studies (75). Justifications for this sample size are based on rationale about feasibility and precision about the mean and variance (75). Both primary and secondary analyses will be used to decide whether the feasibility study should be expanded to a full scale study. The latter implies registering of the trial in accordance the World Health Organisation Trial Registration Data Set (V 1.3.1).

3. Ethics and dissemination

All test procedures involving human participants will be in accordance with the ethical standards of the institutional research committees and with the 1964 Helsinki Declaration and its later amendments. Ethical approval was granted by the Ethics Committee Research UZ/KU Leuven (Registration number B3222024001434) on May 30th 2024. Results will be published in peer-reviewed journals, at scientific conferences, and through press releases.

4. Discussion

4.1 Relevance of the study

For peer review only - http://bmjopen.bmj.com/site/about/guidelines.xhtml

“Maybe not all therapeutic interventions are appropriate for all people with CeH” (12). With this statement Fernández-de-Las-Peñas et al. (2014) drew attention to an important problem in the management of CeH. Others have hypothesized that not all people with CeH can be managed successfully by only targeting the upper-cervical spine (12,13,15). Chua et al. (2011) advised that the management of CeH should not exclusively focus on the cervical nociceptive source, but also on central nervous system sensitization spreading to the level of the trigeminal spinal nucleus (76).

Findings of previous work of our group confirmed this by identifying secondary and tertiary hyperalgesia (i.e. increased pain sensitivity in undamaged tissue away from, and contralateral of the nociceptive source). Meaning that both bilateral cephalic and extra-cephalic PPTs were lower in people with CeH compared to healthy asymptomatic controls (17). However, these findings do not imply that peripheral nociceptive sources should be ignored. Such sources could initiate, maintain, and modulate facilitated central pain processes. Therefore, potential nociceptive sources alongside psychological, behavioural, and social components should also be assessed (i.e. interplay of bottom-up and top-down mechanisms) (77).

Although CPM-testing was already applied to people experiencing various types of headache such as chronic migraine, tension-type headache, and post-traumatic headache (18,78-80), it was only once administered to people with chronic CeH (76). These people with chronic CeH experienced moderate pain (numeric pain rating scale 6.5 ± 1.8) during CPM-testing. We propose to analyse endogenous pain modulation during a headache-free phase. Having a less efficient CPM when being headache-free, suggests that upon a pain-generating event, the person is at a higher risk to develop pain than people showing an efficient CPM at baseline (81).

4.2 The Protocol

We propose a protocol to explore feasibility to assess PMPs in people with CeH. Inter-individual variability in endogenous pain modulation is expected in these people due to their heterogeneous profile (17,60,82,83). Reduced endogenous pain modulation might be expected in people with CeH presenting with modifiable lifestyle and/or psychosocial risk factors for chronification. This hypothesis is supported by PPTs at the suboccipital muscles being influenced by the level of physical activity, stress, quality of life, and screen-time in people with CeH (17). Therefore, we will map the multidimensional PMP (i.e. Psycho-Social Lifestyle Profile and Pain Profile) of each participant. Knowledge of a person’s profile might support an individual management program as opposed to the traditional one-size-fits-all approach (16).

We opted to use a dynamic protocol to assess the Pain Profile of people with CeH rather than relying exclusively on questionnaires and/or static measurements. Caution is indicated when screening questionnaires (e.g. central sensitization inventory) are used to identify human assumed central

For peer review only - http://bmjopen.bmj.com/site/about/guidelines.xhtml

sensitization. These questionnaires are not associated with widespread pain sensitivity, and show a stronger association with psychological measures than with signs of facilitated central pain mechanisms (84).

4.3 Feasibility

Feasibility of the protocol is anticipated since it is designed according to the latest literature in the domain of TSP and CMP measurements (24,52,53,56,66,69,70,85,86). Collaboration with the AZ Vesalius hospitals (Tongeren and Bilzen, Belgium) is foreseen to obtain the required sample size of 12 people with CeH (17,82,83).

A mitigation plan is composed to anticipate potential adverse events related to the testing procedure such as light headache, muscle soreness or sensitivity caused by mechanical pressure on cutaneous, muscular, nervous or arterial tissue. This plan includes general precautions such as informing participants on possible transient adverse effects, applying a strict protocol, using a wide cuff at the least uncomfortable test-location (i.e. calf muscles), and asking to abstain from vigorous physical activity at least 24 hours prior to the measurement. Potential adverse effects are expected to resolve within a 10 minutes recovery period (87). This feasibility study will provide useful information with regard to tolerability, planning, testing study procedures (e.g. estimation of the recruitment rate, plausibility of multicentre collaborations), and investigating outcomes to support adequate sample size calculation (74). Further, such study is designed to ensure that a possible future larger intervention study is achievable, rigorous, and economically justifiable to avoid waste of resources (74). In the current context of a feasibility study, a Data Monitoring Committee will not be involved. Adverse events are expected to be minor and transient in nature, and no long-term follow-up or interventions are applied.

Further, measurements will be conducted with a respected interval (15 minutes after CPM) to limit carry-over of the responses (71,72).

4.4 Limitations and suggestions

We acknowledge that assessing facilitated central pain mechanisms clinically is challenging for several reasons such as the absence of biomarkers to support its diagnosis. Although quantitative sensory testing is a promising surrogate measure to identify such facilitated mechanisms, it only evaluates evoked responses rather than spontaneous pain (84). Results should be interpreted within that context.

More research is needed into relevant modifiable psychosocial and lifestyle factors concerning sensitization of pain in CeH (88). Sedentary-time, anxiety, sleep quality, physical activity, and stress were already reported to be associated with signs of central sensitization in some people with CeH

For peer review only - http://bmjopen.bmj.com/site/about/guidelines.xhtml

(17). Additionally, inter-individual differences explain a large proportion of CPM-variance (89). This finding forces researchers to look at the CeH-population as more heterogeneous (17,60). We propose a feasibility study which includes only 12 participants with CeH, which is the minimum recommended for pilot studies (75).

Further, WUR is reported to lack sensitivity to detect differences in the magnitude of sensitisation. Most studies report little variability (ratio 1.6-1.7) in WUR measures both in normal and hyperalgesic states. Therefore we additionally propose to apply the SumSquare method as described by Allison et al. (2021) (55). The potential future full scale study should analyse endogenous pain modulation within a repeated-measures design to detect variance (i.e. inter-individual differences) (89). Finally, using CPM, TSP, and widespread hyperalgesia to compose the PMP can be time-consuming, expensive, and equipment is often not readily available in clinical practice. Therefore, the current protocol is an essential step towards further research (e.g., assessing feasibility, validity, different populations), including development of easy-to-use alternative valid measurements to be widely implemented in clinical settings.

5. Author's contributions

SM drafted the article. AS, TGN, MG, and WD revised the article critically for essential intellectual and methodological content. All authors approved the version to be published.

6. Competing interest statement

The authors declare no conflict of interest.

7. Funding

AS was funded in whole, or in part, by the Wellcome Trust (222101/Z/20/Z). For the purpose of open access, the author has applied a CC BY public copyright license to any Author Accepted Manuscript version arising from this submission.

TGN is a part of the Center for Neuroplasticity and Pain (CNAP) supported by the Danish National Research Foundation (DNRF121).

SM is supported by the Leuven University (PDMt1/22/016). The sponsor has no role in composing the study design, collection, management, analysis, and interpretation of data, writing of the report, or the decision to submit the report for publication.

For peer review only - http://bmjopen.bmj.com/site/about/guidelines.xhtml

8. References

1. Headache Classification Committee of the International Headache Society (IHS) The International Classification of Headache Disorders, 3rd edition. Cephalalgia 2018;38(1):1-211.
2. Fredriksen TA, Antonaci F, Sjaastad O. Cervicogenic headache: too important to be left un-diagnosed. J Headache Pain 2015;16:6.
3. Kristoffersen ES, Stavem K, Lundqvist C, et al. Impact of chronic headache on workdays, unemployment and disutility in the general population. Journal of Epidemiology and Community Health 2019;73(4):360-7.
4. Raja SN, Carr DB, Cohen M, et al. The revised International Association for the Study of Pain definition of pain: concepts, challenges, and compromises. Pain 2020;161(9):1976-82.
5. Wijma AJ, van Wilgen CP, Meeus M, et al. Clinical biopsychosocial physiotherapy assessment of patients with chronic pain: The first step in pain neuroscience education. Physiother Theory Pract 2016;32(5):368-84.
6. De Pauw R, Dewitte V, de Hertogh W, et al. Consensus among musculoskeletal experts for the management of patients with headache by physiotherapists? A delphi study. Musculoskelet Sci Pract 2021;52:102325.
7. Varatharajan S, Ferguson B, Chrobak K, et al. Are non-invasive interventions effective for the management of headaches associated with neck pain? An update of the Bone and Joint Decade Task Force on Neck Pain and Its Associated Disorders by the Ontario Protocol for Traffic Injury Management (OPTIMa) Collaboration. Eur Spine J 2016;25(7):1971-99.
8. Moore CS, Sibbritt DW, Adams J. A critical review of manual therapy use for headache disorders: prevalence, profiles, motivations, communication and self-reported effectiveness. BMC Neurol. 2017;17(1):61.
9. Gross AR, Paquin JP, Dupont G, et al. Exercises for mechanical neck disorders: A Cochrane review update. Man Ther 2016;24:25-45.
10. Fernandez M, Moore C, Tan J, et al. Spinal manipulation for the management of cervicogenic headache: A systematic review and meta-analysis. Eur J Pain 2020;24(9):1687-702.
11. Coelho M, Ela N, Garvin A, et al. The effectiveness of manipulation and mobilization on pain and disability in individuals with cervicogenic and tension-type headaches: a systematic review and meta-analysis. Phys Ther Rev 2019;24:29-43.
12. Fernández-de-las-Peñas C, Cuadrado ML. Therapeutic options for cervicogenic headache. Expert Rev Neurother 2014;14(1):39-49.
13. Fernández-de-Las-Peñas C, Florencio LL, Plaza-Manzano G, et al. Clinical Reasoning Behind Non-Pharmacological Interventions for the Management of Headaches: A Narrative Literature Review. Int J Environ Res Public Health 2020;17(11):4126.
14. Woolf CJ. Central sensitization: implications for the diagnosis and treatment of pain. Pain 2011;152(3 Suppl):S2-s15.
15. Nijs J, George SZ, Clauw DJ, et al. Central sensitisation in chronic pain conditions: latest discoveries and their potential for precision medicine. The Lancet Rheumatology 2021;3(5):e383-e92.
16. Yarnitsky D, Granot M, Granovsky Y. Pain modulation profile and pain therapy: between pro- and antinociception. Pain 2014;155(4):663-5.
17. Mingels S, Dankaerts W, van Etten L, et al. Exploring multidimensional characteristics in cervicogenic headache: Relations between pain processing, lifestyle, and psychosocial factors. Brain Behav 2021;11(10):e2339.
18. Staud R. Abnormal endogenous pain modulation is a shared characteristic of many chronic pain conditions. Expert Rev Neurother 2012;12(5):577-85.
19. Yarnitsky D, Arendt-Nielsen L, Bouhassira D, et al. Recommendations on terminology and practice of psychophysical DNIC testing. Eur J Pain 2010;14(4):339.
20. Eide PK. Wind-up and the NMDA receptor complex from a clinical perspective. Eur J Pain 2000;4(1):5-15.
21. Price DD, Hayes RL, Ruda M, et al. Neural representation of cutaneous aftersensations by spinothalamic tract neurons. Fed Proc 1978;37(9):2237-9.
22. Arendt-Nielsen L, Morlion B, Perrot S, et al. Assessment and manifestation of central sensitisation across different chronic pain conditions. Eur J Pain 2018;22(2):216-41.
23. Daenen L, Nijs J, Cras P, et al. Changes in Pain Modulation Occur Soon After Whiplash Trauma but are not Related to Altered Perception of Distorted Visual Feedback. Pain Pract 2014;14(7):588-98.
24. Kennedy DL, Kemp HI, Ridout D, et al. Reliability of conditioned pain modulation: a systematic review. Pain 2016;157(11):2410-9.
25. Staud R, Vierck CJ, Cannon RL, et al. Abnormal sensitization and temporal summation of second pain (wind-up) in patients with fibromyalgia syndrome. Pain 2001;91(1-2):165-75.
26. Lacey RJ, Campbell P, Lewis M, et al. The Impact of Inadequate Health Literacy in a Population with Musculoskeletal Pain. Health Lit Res Pract 2018;2(4):e215-e20.
27. Liebert A, Rebbeck T, Elias S, et al. Musculoskeletal physiotherapists' perceptions of non-responsiveness to treatment for cervicogenic headache. Physiother Theory Pract 2013;29(8):616-29.
28. Racine M, Tousignant-Laflamme Y, Kloda LA, et al. A systematic literature review of 10 years of research on sex/gender and pain perception - part 2: do biopsychosocial factors alter pain sensitivity differently in women and men? Pain 2012;153(3):619-35.
29. Yarnitsky D. Role of endogenous pain modulation in chronic pain mechanisms and treatment. Pain 2015;156.

For peer review only - http://bmjopen.bmj.com/site/about/guidelines.xhtml

30. Dhondt E, Danneels L, Van Oosterwijck S, et al. The influence of physical activity on the nociceptive flexion reflex in healthy people. Eur J Pain 2021;25(4):774-89.
31. De Felice M, Ossipov MH. Cortical and subcortical modulation of pain. Pain Management 2016;6(2):111-–20.
32. Bobinski F, Ferreira TAA, Córdova MM, et al. Role of brainstem serotonin in analgesia produced by low-intensity exercise on neuropathic pain after sciatic nerve injury in mice. Pain 2015;156(12):2595-606.
33. Sluka KA, O'Donnell JM, Danielson J, et al. Regular physical activity prevents development of chronic pain and activation of central neurons. J Appl Physiol 2013;114(6):725-33.
34. Stagg NJ, Mata HP, Ibrahim MM, et al. Regular exercise reverses sensory hypersensitivity in a rat neuropathic pain model: role of endogenous opioids. Anesthesiology 2011;114(4):940-8.
35. Staffe AT, Bech MW, Clemmensen SLK, et al. Total sleep deprivation increases pain sensitivity, impairs conditioned pain modulation and facilitates temporal summation of pain in healthy participants. PLoS One 2019;14(12):e0225849.
36. Laugwitz B. Construction and Evaluation of a User Experience Questionnaire. HCI and Usability for Education and Work. Heidelberg: Springer Berlin Heidelberg 2008
37. Learmonth YC, Motl RW. Important considerations for feasibility studies in physical activity research involving persons with multiple sclerosis: a scoping systematic review and case study. Pilot Feasibility Stud 2018;4:1.
38. Thorpe N, Harniess P, Main E, et al. Feasibility, safety and acceptability of select outcome measures in a physiotherapy study protocol for boys with haemophilia. Pilot Feasibility Stud 2021;7(1):105.
39. Mollayeva T, Thurairajah P, Burton K, et al. The Pittsburgh sleep quality index as a screening tool for sleep dysfunction in clinical and non-clinical samples: A systematic review and meta-analysis. Sleep Med Rev 2016;25:52-73.
40. Marinus J, Visser M, van Hilten JJ, et al. Assessment of sleep and sleepiness in Parkinson disease. Sleep 2003;26(8):1049-54.
41. Buysse DJ, Reynolds CF, Monk TH, et al. The Pittsburgh Sleep Quality Index: a new instrument for psychiatric practice and research. Psychiatry Res 1989;28(2):193-213.
42. Smyth CA. Evaluating sleep quality in older adults: the Pittsburgh Sleep Quality Index can be used to detect sleep disturbances or deficits. Am J Nurs 2008;108(5):42-50; quiz -1.
43. Parkitny L, McAuley JH, Walton D, et al. Rasch analysis supports the use of the depression, anxiety, and stress scales to measure mood in groups but not in individuals with chronic low back pain. J Clin Epidemiol 2012;65(2):189-98.
44. de Beurs E, Van Dyck R, Marquenie LA, et al. De DASS: Een vragenlijst voor het meten van depressie, angst en stress. [The DASS: A questionnaire for the measurement of depression, anxiety, and stress.]. Gedragstherapie 2001;34(1):35-53.
45. Lovibond PF, Lovibond SH. The structure of negative emotional states: comparison of the Depression Anxiety Stress Scales (DASS) with the Beck Depression and Anxiety Inventories. Behav Res Ther 1995;33(3):335-43.
46. Buse DC, Sollars CM, Steiner TJ, et al. Why HURT? A review of clinical instruments for headache management. Curr Pain Headache Rep 2012;16(3):237-54.
47. Martin M, Blaisdell B, Kwong JW, et al. The Short-Form Headache Impact Test (HIT-6) was psychometrically equivalent in nine languages. J Clin Epidemiol 2004;57(12):1271-8.
48. Kawata AK, Coeytaux RR, Devellis RF, et al. Psychometric properties of the HIT-6 among patients in a headache-specialty practice. Headache 2005;45(6):638-43.
49. Kosinski M, Bayliss MS, Bjorner JB, et al. A six-item short-form survey for measuring headache impact: the HIT-6. Qual Life Res 2003;12(8):963-74.
50. Moana-Filho EJ, Herrero Babiloni A. Endogenous pain modulation in chronic temporomandibular disorders: Derivation of pain modulation profiles and assessment of its relationship with clinical characteristics. J Oral Rehabil 2019;46(3):219-32.
51. Gil-Martínez A, Grande-Alonso M, La Touche R, et al. Psychosocial and Somatosensory Factors in Women with Chronic Migraine and Painful Temporomandibular Disorders. Pain Res Manag 2016;2016:3945673.
52. Rolke R, Magerl W, Campbell KA, et al. Quantitative sensory testing: a comprehensive protocol for clinical trials. Eur J Pain 2006;10(1):77-88.
53. Rolke R, Baron R, Maier C, et al. Quantitative sensory testing in the German Research Network on Neuropathic Pain (DFNS): standardized protocol and reference values. Pain 2006;123(3):231-43.
54. Baeumler PI, Conzen P, Irnich D. High Temporal Summation of Pain Predicts Immediate Analgesic Effect of Acupuncture in Chronic Pain Patients-A Prospective Cohort Study. Front Neurosci 2019;13:498.
55. Allison C, Korey L, John ZS. A novel computational technique for the quantification of temporal summation in healthy individuals. Musculoskelet Sci Pract 2021;54:102400.
56. Marcuzzi A, Wrigley PJ, Dean CM, et al. The long-term reliability of static and dynamic quantitative sensory testing in healthy individuals. Pain 2017;158(7):1217-23.
57. Bogduk N. The neck and headaches. Neurol Clin 2014;32(2):471-87.
58. Alburquerque-Sendín F, Madeleine P, Fernández-de-Las-Peñas C, et al. Spotlight on topographical pressure pain sensitivity maps: a review. J Pain Res 2018;11:215-25.
59. Walton DM, Macdermid JC, Nielson W, et al. Reliability, standard error, and minimum detectable change of clinical pressure pain threshold testing in people with and without acute neck pain. J Orthop Sports Phys Ther 2011;41(9):644-50.
60. Mingels S, Dankaerts W, Bruckers L, et al. Inter-individual variability in mechanical pain sensation in patients with cervicogenic headache: an explorative study. Sci Rep 2022;12(1):20635.
61. Vaegter HB, Graven-Nielsen T. Pain modulatory phenotypes differentiate subgroups with different clinical and experimental pain sensitivity. Pain 2016;157(7):1480-8.

For peer review only - http://bmjopen.bmj.com/site/about/guidelines.xhtml

62. Ylinen J, Nykänen M, Kautiainen H, et al. Evaluation of repeatability of pressure algometry on the neck muscles for clinical use. Man Ther 2007;12(2):192-7.
63. Zicarelli CAM, Santos JPM, Poli-Frederico RC, et al. Reliability of pressure pain threshold to discriminate individuals with neck and low back pain. J Back Musculoskelet Rehabil 2021;34(3):363-70.
64. Cummins TM, Kucharczyk MM, Graven-Nielsen T, et al. Activation of the descending pain modulatory system using cuff pressure algometry: Back translation from man to rat. Eur J Pain 2020;24(7):1330-8.
65. Graven-Nielsen T, Vaegter HB, Finocchietti S, et al. Assessment of musculoskeletal pain sensitivity and temporal summation by cuff pressure algometry: a reliability study. Pain 2015;156(11):2193-202.
66. Hoegh M, Petersen KK, Graven-Nielsen T. Effects of repeated conditioning pain modulation in healthy volunteers. Eur J Pain. 2018;22(10):1833-43.
67. Inventors'Way ApS. Cuff Pressure Algometry [online]. 2023. https://nocitech.com (accessed April 15th 2023).
68. Kvistgaard Olsen J, Fener DK, Wæhrens EE, et al. Reliability of Pain Measurements Using Computerized Cuff Algometry: A DoloCuff Reliability and Agreement Study. Pain Pract 2017;17(6):708-17.
69. Lindskou TA, Christensen SW, Graven-Nielsen T. Cuff Algometry for Estimation of Hyperalgesia and Pain Summation. Pain Med 2017;18(3):468-76.
70. Graven-Nielsen T, Izumi M, Petersen KK, et al. User-independent assessment of conditioning pain modulation by cuff pressure algometry. Eur J Pain 2017;21(3):552-61.
71. Vaegter HB, Handberg G, Graven-Nielsen T. Similarities between exercise-induced hypoalgesia and conditioned pain modulation in humans. Pain 2014;155(1):158-67.
72. Vaegter HB, Handberg G, Jørgensen MN, et al. Aerobic exercise and cold pressor test induce hypoalgesia in active and inactive men and women. Pain Med 2015;16(5):923-33.
73. Graven-Nielsen T, Arendt-Nielsen L. Assessment of mechanisms in localized and widespread musculoskeletal pain. Nat Rev Rheumatol 2010;6(10):599-606.
74. Thabane L, Ma J, Chu R, et al. A tutorial on pilot studies: the what, why and how. BMC Med Res Methodol 2010;10:1.
75. Julious SA. Sample size of 12 per group rule of thumb for a pilot study. Pharmaceutical Statistics: The Journal of Applied Statistics in the Pharmaceutical Industry 2005;4(4):287-91.
76. Chua NH, van Suijlekom HA, Vissers KC, et al. Differences in sensory processing between chronic cervical zygapophysial joint pain patients with and without cervicogenic headache. Cephalalgia 2011;31(8):953-63.
77. van Griensven H, Schmid A, Trendafilova T, et al. Central Sensitization in Musculoskeletal Pain: Lost in Translation? J Orthop Sports Phys Ther 2020;50(11):592-6.
78. Nahman-Averbuch H, Granovsky Y, Coghill RC, et al. Waning of "conditioned pain modulation": a novel expression of subtle pronociception in migraine. Headache 2013;53(7):1104-15.
79. Defrin R, Riabinin M, Feingold Y, et al. Deficient pain modulatory systems in patients with mild traumatic brain and chronic post-traumatic headache: implications for its mechanism. J Neurotrauma 2015;32(1):28-37.
80. Exposto FG, Bendixen KH, Ernberg M, et al. Assessment of Pain Modulatory and Somatosensory Profiles in Chronic Tension-Type Headache Patients. Pain Med 2021;22(10):2356-65.
81. Yarnitsky D. Role of endogenous pain modulation in chronic pain mechanisms and treatment. Pain 2015;156:S24-S31.
82. Mingels S, Dankaerts W, van Etten L, et al. Spinal postural variability relates to biopsychosocial variables in patients with cervicogenic headache. Sci Rep 2021;11(1):13783.
83. Mingels S, Dankaerts W, van Etten L, et al. Lower spinal postural variability during laptop-work in subjects with cervicogenic headache compared to healthy controls. Sci Rep 2021;11(1):5159.
84. Coronado RA, George SZ. The Central Sensitization Inventory and Pain Sensitivity Questionnaire: An exploration of construct validity and associations with widespread pain sensitivity among individuals with shoulder pain. Musculoskelet Sci Pract 2018;36:61-7.
85. Vaegter HB, Petersen KK, Mørch CD, et al. Assessment of CPM reliability: quantification of the within-subject reliability of 10 different protocols. Scand J Pain 2018;18(4):729-37.
86. Costa YM, de Araújo-Júnior ENS, Fiedler LS, et al. Reproducibility of quantitative sensory testing applied to musculoskeletal orofacial region: Site and sex differences. Eur J Pain 2019;23(1):81-90.
87. Langham MC, Floyd TF, Mohler ER, et al. Evaluation of cuff-induced ischemia in the lower extremity by magnetic resonance oximetry. J Am Coll Cardiol 2010;55(6):598-606.
88. Nahman-Averbuch H, Nir RR, Sprecher E, et al. Psychological Factors and Conditioned Pain Modulation: A Meta-Analysis. Clin J Pain 2016;32(6):541-54.
89. Graeff P, Itter A, Wach K, et al. Inter-Individual Differences Explain More Variance in Conditioned Pain Modulation Than Age, Sex and Conditioning Stimulus Intensity Combined. Brain Sci 2021;11(9)1186.

For peer review only - http://bmjopen.bmj.com/site/about/guidelines.xhtml

9. Figure legend

Figure 1. Visualisation of the composition of the PMP (PMP = Pain Modulation Profile; CPM = Conditioned Pain Modulation; TSP = Temporal Summation of Pain; PPT = Pressure Pain Threshold).

Figure 2. Visualisation of the experimental protocol regarding TSP (PP = PinPrick; V1 = ophthalmic branch trigeminal nerve; min = minutes).

Figure 3. Visualisation of the experimental protocol regarding CPM (PPT = Pressure Pain Threshold (kPa/cm²); PPTo = Pressure Pain Tolerance (kPa/cm²); Sub = Suboccipital; L = Left; R = Right; CPA = Cuff Pressure Algometry non-dominant calf muscle (kPa); s = seconds; min = minutes).

Figure 4. Visualisation of the experimental protocol regarding widespread pain (PPT = Pressure Pain Threshold (kPa/cm²); Sub = Suboccipital; L = Left; R = Right; Tib = Tibialis anterior).

For peer review only

For peer review only - http://bmjopen.bmj.com/site/about/guidelines.xhtml

FIGURE 1

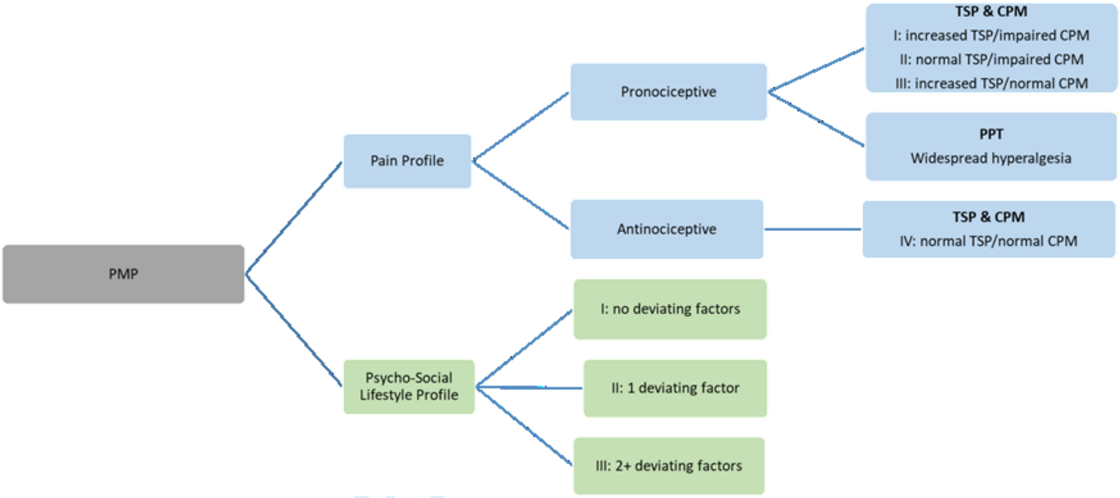

Figure 1. Visualisation of the composition of the PMP (PMP = Pain Modulation Profile; CPM = Conditioned Pain Modulation; TSP = Temporal Summation of Pain; PPT = Pressure Pain Threshold).

FIGURE 2

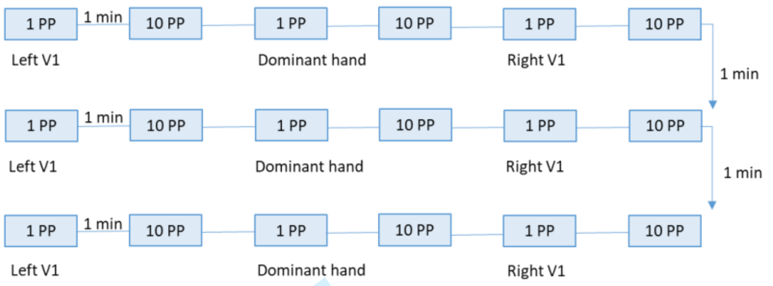

Figure 2. Visualisation of the experimental protocol regarding TSP (PP = PinPrick; V1 = ophthalmic branch trigeminal nerve; min = minutes).

For peer review only

FIGURE 3

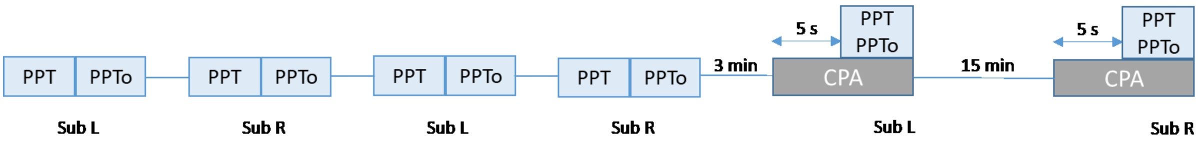

Figure 3. Visualisation of the experimental protocol regarding CPM (PPT = Pressure Pain Threshold (kPa/cm²); PPTo = Pressure Pain Tolerance (kPa/cm²); Sub = Suboccipital; L = Left; R = Right; CPA = Cuff Pressure Algometry non-dominant calf muscle (kPa); s = seconds; min = minutes).

For peer review only

FIGURE 4

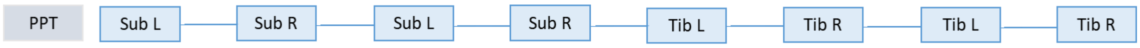

Figure 4. Visualisation of the experimental protocol regarding widespread pain (PPT = Pressure Pain Threshold (kPa/cm²); Sub = Suboccipital; L = Left; R = Right; Tib = Tibialis anterior).

For peer review only

SUPPLEMENTARY FILE

Supplement 1 – Checklist participant recruitment

The criteria that will be used to in- and exclude participants are summarized in table S1.

Table S1. Summary of inclusion- and exclusion criteria for participants with CeH.

**Inclusion**	Caucasian males, females between 18-55 years
	Dutch-speaking
	Fulfilment of the diagnostic criteria for secondary CeH conform the ICHD-3 or CHISG (1,2)
	Diagnostic conformation by a neurologist
	Normal cognitive capacity (Mini Mental State Examination test score of 30).
	Willing to participate
**Exclusion**	Any other headache type or headache-related disorder
	Participation in another study related to headache
	Pregnancy
	Smoking
	First headache onset > 50 years
	Spinal surgery, head/neck trauma, headache-related physiotherapy ≤ 4 weeks prior the study
	Post-dural punction 2 weeks prior to the study
	Cognitive limitations (Mini Mental State Examination test score < 30)
	Confounding pathologies
	- Musculoskeletal: hernia, disk prolapse, congenital spinal deviations, TMD, hypermobility (Beighton ≥ 4)
	- Neurological: MS, Parkinson, epilepsy, CVA, myelopathy, myopathy, Benign Paroxysmal Position Vertigo, neurodegeneration, meningitis, encephalitis, dystonia, visual disorder
	- Endocrine - System: FM, CFS, rheumatoid arthritis, infectious/inflammatory diseases, sinusitis
	- Vascular: dysregulation blood pressure, postural hypotension, vertebra-basilar symptoms
	- Psychiatric
	- Medication-overuse: ergotamine, NSAID's, opioids, acetylsalicylic acid, triptans, simple analgesics (> 10 days/month for > 3 months)
	- Withdrawal of alcohol, caffeine or medication
	- Other: orthodontics, sleep apnoea

ICHD = International Classification Headache Disorders; CHISG = Cervicogenic Headache International Study Group Diagnostic; NSAID = Non Steroid Anti Inflammatory; MS = Multiple Sclerosis; CVA = Cerebro-Vascular Accident; FM = Fibromyalgia; CFS = Chronic Fatigue Syndrome; TMD = Temporo-mandibular disorder.

For peer review only

For peer review only - http://bmjopen.bmj.com/site/about/guidelines.xhtml

Supplement 2 – Time schedule

The time schedule for the feasibility study is visualized via figure S1.

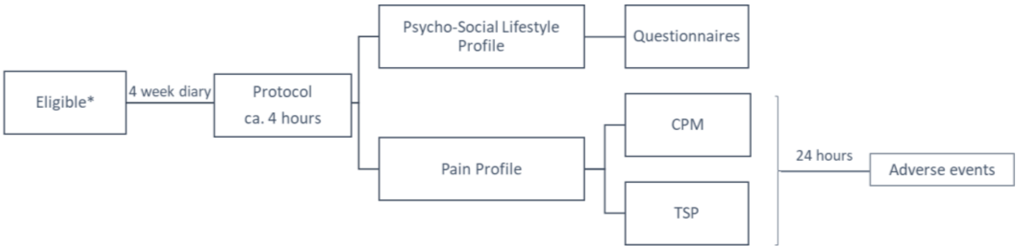

Figure S1. Visualisation of the time schedule for the feasibility study (* = estimated time to recruit at least 12 people with CeH = 3 months based on previous experience; CPM = Conditioned Pain Modulation; TSP = Temporal Summation of Pain).

For peer review only - http://bmjopen.bmj.com/site/about/guidelines.xhtml

Supplement 3 – Conditioned pain modulation – widespread hyperalgesia

Figure S2 provides a summary of the second CPM protocol.

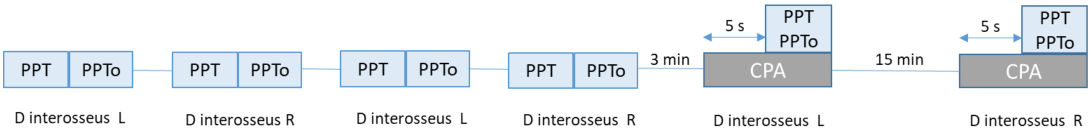

Figure S2. Visualisation of the experimental protocol regarding CPM (PPT = Pressure Pain Threshold (kPa/cm²); PPTo = Pressure Pain Tolerance (kPa/cm²); D = Dorsal; L = Left; R = Right; CPA = Cuff Pressure Algometry non-dominant calf muscle (kPa); s = seconds; min = minutes).

Test stimulus: Mechanical pressure pain thresholds and tolerance

Measurements will be performed 15 minutes after the CPM with the testing stimulus applied at the suboccipital muscles. Pressure pain threshold (PPT) and pressure pain tolerance (PPTo) (kPa/cm²) will be measured at the left and right muscle belly of the first dorsal interosseous muscle using an electronic pressure algometer (Somedic AB, Stockholm, Sweden) (3,4). Pressure will be perpendicularly applied directly on the midpoint of the muscle belly, starting at 0 to maximal 1000 kPa, using a 1 cm² probe with a slope of 30 kPa/s. (5,6). Participants will be instructed to push the stop-button when the sensation of pressure first elicits pain (PPT), and when the maximum amount of pressure that could be tolerated is reached (PPTo).Intrarater reliability of the first dorsal interosseous muscle is excellent in healthy participants (ICC 0.91) (3).

Two repeated measurements at baseline, and two measurements parallel with the conditioning stimulus will be performed (figure S2). Averages of the extracted parameters will be used for further analysis (7).

Conditioning stimulus: Cuff pressure algometry

Cuff pressure tolerance (kPa) will be measured at the non-dominant lower leg. A cuff (Nocitech, Aalborg University, Denmark) will be placed around the bare non-dominant calf muscle, mounted with 8-cm distance between its upper border and the tibial tuberosity (8,9). Upper and lower borders of the cuff will be marked on the skin to ensure the cuff does not move between stimulations. Pressure tolerance will be determined through the maximal amount of pressure that can be tolerated. Intensity of the cuff pressure for the conditioning stimulus will be predefined as 70% of the pressure tolerance on the non-dominant leg (9,10). Computer-controlled cuff algometry (LabBench® CPAR+ instrument) shows an excellent intra-rater reliability (ICC 0.89) in healthy subjects (11,12). Test-retest reliability is good (ICCs 0.74 – 0.87) in healthy subjects (8).

For peer review only - http://bmjopen.bmj.com/site/about/guidelines.xhtml

Conditioned pain modulation (CPM)

CPM will be assessed 3 minutes after determining cuff pressure tolerance. The pressure (conditioning stimulus) will be kept constant throughout the CPM protocol (9,10). Five seconds after inflation of the cuff, PPT and PPTo will be reassessed as described above. Participants will be informed that the conditioning stimulus will be moderately painful, but that they should focus their attention on the test stimulus. The procedure described above will be sequentially and twice executed, i.e. once with the PPT-measurement at the left first dorsal interosseous muscle, and once with the PPT-measurement at the right first dorsal interosseous muscle as test stimulus. A 15-minute-interval will be provided between the CPM-measurements since CPM has a short lasting (< 15 minutes) hypoalgesic effect (figure S2) (14,15).

At the moment, there is no consensus on a normal CPM effect. We propose a method described by Vaegter et al. (2016) to categorize the CPM response as normal or impaired based on the within-subject coefficient of variation (= within-subject SD/within-subject mean) in PPT (7). Participants will be profiled as having an impaired CPM if the CPM response is less than or equal to the normal within-subject coefficient of variation in PPT between 2 repeated assessments, and as having normal CPM if the CPM response is greater than the normal variation plus the upper limit of the 95% confidence interval (7).

For peer review only

Supplement 4 – Summary of the primary outcomes

Table S2 provides a summary of the primary outcomes to estimate feasibility.

Table S2. Summary of the adapted feasibility metrics and objectives (15,16).

Metric	Objectives	Outcome
**Process**	Determine recruitment and/or refusal rates per month	Percentage
Assess feasibility of processes key for future studies	Determine premature session endings and reasons(s) (e.g. pain, discomfort)	Percentage
	Operational feasibility (User Experience Questionnaire on usefulness, satisfaction, ease of learning, ease of use, and user experience after testing) (17)	Scores on a 7-point Likert scale converted to values ranging between < -3 (bad evaluation) and > 3 (extremely good evaluation)
**Resource**	Demonstrate appropriate eligibility criteria	Percentage and Qualitative
Assess time and resource problems that might occur in future studies	Estimate barriers/refusals to participation	Percentage and Qualitative
	Estimate access to/cost of equipment, space, time	Estimate cost/participant (i.e. staff, equipment, facility overheads)
	Clinician training needs and competence	Staff training requirements (time, frequency)
**Management**	Estimate equipment usage (e.g. availability)	Qualitative
Assess potential human and data management problems	Determine processing time for data collection	Time to mail data collection, time to complete outcome assessment
	Data collection and analysis – software appropriateness	Qualitative (identify requirements)
**Scientific**	Estimate challenges perceived/experienced by researcher	Qualitative
Assess safety, burden, data collection and response	Determine adverse events and safety procedure (e.g. local pain)	Percentage and Qualitative
	Burden of research	Percentage and Qualitative (feedback from participants before/during/after measurements)
	Determine appropriateness of target group	Qualitative (feedback from participants)
	Determine acceptability to participants	Qualitative (feedback from participants)
	Estimate outcome measures SD to determine future sample size	Statistical analysis of CPM (Pain Profile) to estimate future sample size

CPM = Conditioned Pain Modulation; SD = Standard Deviation.

For peer review only

For peer review only - http://bmjopen.bmj.com/site/about/guidelines.xhtml

References

1. Headache Classification Committee of the International Headache Society (IHS) The International Classification of Headache Disorders, 3rd edition. Cephalalgia 2018;38(1):1-211.
2. Fredriksen TA, Antonaci F, Sjaastad O. Cervicogenic headache: too important to be left un-diagnosed. J Headache Pain 2015;16:6.
3. Chesterton LS, Sim J, Wright CC, et al. Interrater reliability of algometry in measuring pressure pain thresholds in healthy humans, using multiple raters. Clin J Pain 2007;23(9):760-6.
4. Bogduk N. The neck and headaches. Neurol Clin 2014;32(2):471-87.
5. Alburquerque-Sendín F, Madeleine P, Fernández-de-Las-Peñas C, et al. Spotlight on topographical pressure pain sensitivity maps: a review. J Pain Res 2018;11:215-25.
6. Mingels S, Dankaerts W, van Etten L, et al. Exploring multidimensional characteristics in cervicogenic headache: Relations between pain processing, lifestyle, and psychosocial factors. Brain Behav 2021;11(10):e2339.
7. Vaegter HB, Graven-Nielsen T. Pain modulatory phenotypes differentiate subgroups with different clinical and experimental pain sensitivity. Pain 2016;157(7):1480-8.
8. Graven-Nielsen T, Vaegter HB, Finocchietti S, et al. Assessment of musculoskeletal pain sensitivity and temporal summation by cuff pressure algometry: a reliability study. Pain 2015;156(11):2193-202.
9. Cummins TM, Kucharczyk MM, Graven-Nielsen T, et al. Activation of the descending pain modulatory system using cuff pressure algometry: Back translation from man to rat. Eur J Pain 2020;24(7):1330-8.
10. Hoegh M, Petersen KK, Graven-Nielsen T. Effects of repeated conditioning pain modulation in healthy volunteers. Eur J Pain. 2018;22(10):1833-43.
11. Inventors'Way ApS. Cuff Pressure Algometry [online]. 2023. https://nocitech.com (accessed April 15th 2023).
12. Kvistgaard Olsen J, Fener DK, Wæhrens EE, et al. Reliability of Pain Measurements Using Computerized Cuff Algometry: A DoloCuff Reliability and Agreement Study. Pain Pract 2017;17(6):708-17.
13. Vaegter HB, Handberg G, Graven-Nielsen T. Similarities between exercise-induced hypoalgesia and conditioned pain modulation in humans. Pain 2014;155(1):158-67.
14. Vaegter HB, Handberg G, Jørgensen MN, et al. Aerobic exercise and cold pressor test induce hypoalgesia in active and inactive men and women. Pain Med 2015;16(5):923-33.
15. Learmonth YC, Motl RW. Important considerations for feasibility studies in physical activity research involving persons with multiple sclerosis: a scoping systematic review and case study. Pilot Feasibility Stud 2018;4:1.
16. Thorpe N, Harniess P, Main E, et al. Feasibility, safety and acceptability of select outcome measures in a physiotherapy study protocol for boys with haemophilia. Pilot Feasibility Stud 2021;7(1):105.
17. Laugwitz B. Construction and Evaluation of a User Experience Questionnaire. HCI and Usability for Education and Work. Heidelberg: Springer Berlin Heidelberg 2008

For peer review only

For peer review only - http://bmjopen.bmj.com/site/about/guidelines.xhtml

SPIRIT CHECKLIST

Table 1. SPIRIT 2013 Checklist: Recommended items to address in a clinical trial protocol and related documents (1)*

Section/item	Item	Description	Page
**Administrative information**			
Title	1	Descriptive title identifying the study design, population, interventions, and, if applicable, trial acronym	1
Trial registration	2a	Trial identifier and registry name. If not yet registered, name of intended registry	13
	2b	All items from the World Health Organization Trial Registration Data Set	13
Protocol version	3	Date and version identifier	1
Funding	4	Sources and types of financial, material, and other support	16
Roles and responsibilities	5a	Names, affiliations, and roles of protocol contributors	1,16
	5b	Name and contact information for the trial sponsor	16
	5c	Role of study sponsor and funders, if any, in study design; collection, management, analysis, and interpretation of data; writing of the report; and the decision to submit the report for publication, including whether they will have ultimate authority over any of these activities	16
	5d	Composition, roles, and responsibilities of the coordinating centre, steering committee, endpoint adjudication committee, data management team, and other individuals or groups overseeing the trial, if applicable (see Item 21a for data monitoring committee)	n/a
**Introduction**			
Background and rationale	6a	Description of research question and justification for undertaking the trial, including summary of relevant studies (published and unpublished) examining benefits and harms for each intervention	3,4
	6b	Explanation for choice of comparators	n/a
Objectives	7*	Specific objectives or research questions for pilot trial	4
Trial design	8	Description of trial design including type of trial (eg, parallel group, crossover, factorial, single group), allocation ratio, and framework (eg, superiority, equivalence, noninferiority, exploratory)	4,5
**Methods: Participants, interventions, and outcomes**			
Study setting	9	Description of study settings (eg, community clinic, academic hospital) and list of countries where data will be collected. Reference to where list of study sites can be obtained	5,6
Eligibility criteria	10	Inclusion and exclusion criteria for participants. If applicable, eligibility criteria for study centres and individuals who will perform the interventions (eg, surgeons, psychotherapists)	5, Appendix A
Interventions	11a	Interventions for each group with sufficient detail to allow replication, including how and when they will be administered	6,7 / 9-12 / Appendix B Figures 1-4
	11b	Criteria for discontinuing or modifying allocated interventions for a given trial participant (eg, drug dose change in response to harms, participant request, or improving/worsening disease)	6,8

For peer review only - http://bmjopen.bmj.com/site/about/guidelines.xhtml

	11c	Strategies to improve adherence to intervention protocols, and any procedures for monitoring adherence (eg, drug tablet return, laboratory tests)	n/a
	11d	Relevant concomitant care and interventions that are permitted or prohibited during the trial	6
Outcomes	12*	Completely defined prespecified assessments or measurements to address each pilot trial objective, including how and when they were assessed	6-7 / 9-12 / Appendix B
Participant timeline	13	Time schedule of enrolment, interventions (including any run-ins and washouts), assessments, and visits for participants. A schematic diagram is highly recommended (see Figure)	6, Appendix C
Sample size	14*	Rationale for numbers in the pilot trial	8,13
Recruitment	15	Strategies for achieving adequate participant enrolment to reach target sample size	14,15
**Methods: Assignment of interventions (for controlled trials)**			
Allocation:			
Sequence generation	16a	Method of generating the allocation sequence (eg, computer-generated random numbers), and list of any factors for stratification. To reduce predictability of a random sequence, details of any planned restriction (eg, blocking) should be provided in a separate document that is unavailable to those who enrol participants or assign interventions	n/a
Allocation concealment mechanism	16b	Mechanism of implementing the allocation sequence (eg, central telephone; sequentially numbered, opaque, sealed envelopes), describing any steps to conceal the sequence until interventions are assigned	n/a
Implementation	16c	Who will generate the allocation sequence, who will enrol participants, and who will assign participants to interventions	n/a
Blinding (masking)	17a	Who will be blinded after assignment to interventions (eg, trial participants, care providers, outcome assessors, data analysts), and how	n/a
	17b	If blinded, circumstances under which unblinding is permissible, and procedure for revealing a participant's allocated intervention during the trial	n/a
**Methods: Data collection, management, and analysis**			
Data collection methods	18a	Plans for assessment and collection of outcome, baseline, and other trial data, including any related processes to promote data quality (eg, duplicate measurements, training of assessors) and a description of study instruments (eg, questionnaires, laboratory tests) along with their reliability and validity, if known. Reference to where data collection forms can be found, if not in the protocol	6-12
	18b	Plans to promote participant retention and complete follow-up, including list of any outcome data to be collected for participants who discontinue or deviate from intervention protocols	5,6,8
Data management	19	Plans for data entry, coding, security, and storage, including any related processes to promote data quality (eg, double data entry; range checks for data values). Reference to where details of data management procedures can be found, if not in the protocol	12,13
Statistical methods	20a*	Methods used to address each pilot trial objective whether qualitative or quantitative	13

For peer review only - http://bmjopen.bmj.com/site/about/guidelines.xhtml

	20b	Methods for any additional analyses (eg, subgroup and adjusted analyses)	n/a
	20c	Definition of analysis population relating to protocol non-adherence (eg, as randomised analysis), and any statistical methods to handle missing data (eg, multiple imputation)	n/a
**Methods: Monitoring**			
Data monitoring	21a	Composition of data monitoring committee (DMC); summary of its role and reporting structure; statement of whether it is independent from the sponsor and competing interests; and reference to where further details about its charter can be found, if not in the protocol. Alternatively, an explanation of why a DMC is not needed	15
	21b	Description of any interim analyses and stopping guidelines, including who will have access to these interim results and make the final decision to terminate the trial	n/a
Harms	22	Plans for collecting, assessing, reporting, and managing solicited and spontaneously reported adverse events and other unintended effects of trial interventions or trial conduct	5,6,8
Auditing	23	Frequency and procedures for auditing trial conduct, if any, and whether the process will be independent from investigators and the sponsor	n/a
**Ethics and dissemination**			
Research ethics approval	24	Plans for seeking research ethics committee/institutional review board (REC/IRB) approval	5
Protocol amendments	25	Plans for communicating important protocol modifications (eg, changes to eligibility criteria, outcomes, analyses) to relevant parties (eg, investigators, REC/IRBs, trial participants, trial registries, journals, regulators)	6
Consent or assent	26a	Who will obtain informed consent or assent from potential trial participants or authorised surrogates, and how (see Item 32)	5
	26b	Additional consent provisions for collection and use of participant data and biological specimens in ancillary studies, if applicable	n/a
Confidentiality	27	How personal information about potential and enrolled participants will be collected, shared, and maintained in order to protect confidentiality before, during, and after the trial	12,13
Declaration of interests	28	Financial and other competing interests for principal investigators for the overall trial and each study site	16
Access to data	29	Statement of who will have access to the final trial dataset, and disclosure of contractual agreements that limit such access for investigators	12,13
Ancillary and post-trial care	30	Provisions, if any, for ancillary and post-trial care, and for compensation to those who suffer harm from trial participation	n/a
Dissemination policy	31a	Plans for investigators and sponsor to communicate trial results to participants, healthcare professionals, the public, and other relevant groups (eg, via publication, reporting in results databases, or other data sharing arrangements), including any publication restrictions	n/a
	31b	Authorship eligibility guidelines and any intended use of professional writers	n/a
	31c	Plans, if any, for granting public access to the full protocol, participant-level dataset, and statistical code	n/a

For peer review only - http://bmjopen.bmj.com/site/about/guidelines.xhtml

**Appendices**			
Informed consent materials	32	Model consent form and other related documentation given to participants and authorised surrogates	Appendix D
Biological specimens	33	Plans for collection, laboratory evaluation, and storage of biological specimens for genetic or molecular analysis in the current trial and for future use in ancillary studies, if applicable	n/a

n/a = Not Applicable; * = item numbers 12, 14, and 20a were replaced by items 6a, 7a, and 12a from the CONSORT-extensions (2) checklist based on the article of Thabane and Lancaster (2019) (3)

REFERENCES

1. Chan A-W, Tetzlaff JM, Altman DG, et al. SPIRIT 2013 Statement: Defining standard protocol items for clinical trials. Ann Intern Med 2013;158(3):200-7.
2. Eldridge SM, Chan CL, Campbell MJ, et al. CONSORT 2010 statement: extension to randomised pilot and feasibility trials. BMJ 2016;355:i5239.
3. Thabane, L., Lancaster, G. A guide to the reporting of protocols of pilot and feasibility trials. Pilot Feasibility Stud 2019;5:37.

For peer review only
